# BA9 lineage of respiratory syncytial virus from across the globe and its evolutionary dynamics

**DOI:** 10.1371/journal.pone.0193525

**Published:** 2018-04-25

**Authors:** Md Shakir Hussain Haider, Wajihul Hasan Khan, Farah Deeba, Sher Ali, Anwar Ahmed, Irshad H. Naqvi, Ravins Dohare, Hytham A. Alsenaidy, Abdulrahman M. Alsenaidy, Shobha Broor, Shama Parveen

**Affiliations:** 1 Centre for Interdisciplinary Research in Basic Sciences, Jamia Millia Islamia, New Delhi, India; 2 Kusuma School of Biological Sciences, Indian Institute of Technology Delhi, New Delhi, India; 3 Department of Biochemistry, College of Science, King Saud University, Riyadh, Saudi Arabia; 4 Centre for Excellence in Biotechnology Research, Department of Biochemistry, College of Science, King Saud University, Riyadh, Saudi Arabia; 5 Dr. M. A. Ansari Health Centre, Jamia Millia Islamia, New Delhi, India; 6 College of Medicine, Shaqra University, Shaqra, Saudi Arabia; 7 Department of Microbiology, Faculty of Medicine and Health Science, Shree Guru Gobind Singh Tricentenary University, Gurgaon, Haryana, India; Monash University, Australia, AUSTRALIA

## Abstract

Respiratory syncytial virus (RSV) is an important pathogen of global significance. The BA9 is one of the most predominant lineages of the BA genotype of group B RSV that has acquired a 60bp duplication in its G protein gene. We describe the local and global evolutionary dynamics of the second hyper variable region in the C- terminal of the G protein gene of the BA9 lineage. A total of 418 sequences (including 31 study and 387 GenBank strains) from 29 different countries were used for phylogenetic analysis. This analysis showed that the study strains clustered with BA (BA9 and BA8) and SAB4 genotype of group B RSV. We performed time-scaled evolutionary clock analyses using Bayesian Markov chain Monte Carlo methods. We also carried out glycosylation, selection pressure, mutational, entropy and Network analyses of the BA9 lineage. The time to the most recent common ancestor (tMRCA) of the BA genotype and BA9 lineage were estimated to be the years 1995 (95% HPD; 1987–1997) and 2000 (95% HPD; 1998–2001), respectively. The nucleotide substitution rate of the BA genotype [(4.58×10^−3^ (95% HPD; 3.89–5.29×10^−3^) substitution/site/year] was slightly faster than the BA9 lineage [4.03×10^−3^ (95% HPD; 4.65–5.2492×10^−3^)]. The BA9 lineage was categorized into 3 sub lineages (I, II and III) based on the Bayesian and Network analyses. The local transmission pattern suggested that BA9 is the predominant lineage of BA viruses that has been circulating in India since 2002 though showing fluctuations in its effective population size. The BA9 lineage established its global distribution with report from 23 different countries over the past 16 years. The present study augments our understanding of RSV infection, its epidemiological dynamics warranting steps towards its overall global surveillance.

## Introduction

Respiratory Syncytial Virus (RSV) is the most frequent respiratory pathogen of severe respiratory tract infection in infants and children worldwide and is the major cause of hospitalization [1]. RSV is an enveloped virus with a non-segmented, negative-sense, single stranded RNA genome of approximately 15.2 Kb in length. The G protein is a type II integral trans-membrane glycoprotein that shows frequent divergence at the nucleotide and amino acid levels [2]. Two antigenic groups of RSV (A and B) have been identified based on the cross-reactivity of antibodies with the G protein [3, 4]. Both the groups were categorized into several genotypes on the basis of genetic divergence within the second hypervariable region (2^nd^ HVR) of G protein gene. Group A have been categorized as GA1–GA7, SAA1-SAA2, NA1–NA4 and ON1 genotypes [5–11]. Group B includes, GB1–GB6, SAB1–SAB4 and BA1–BA12 genotypes [5, 7, 12–17]. More than one genotypes or lineages of both the groups can co-circulate in a community, while new genotypes or lineages can dominate over the previous ones [18].

A new genotype of RSV was identified in Buenos Aires, Argentina in 1999 with a duplication of 60bp in the 2^nd^ HVR of G protein gene [19]. This region plays an important role accommodating selective pressure during the course of evolution. Consequently, it results in the enhanced fitness of the viral population leading to its efficient global transmission including detection in India [18, 20–25]. The first report of BA genotype from New Delhi, India was described by our group during the years 2001–05 [26]. Further, an investigation from Kolkata, West Bengal during the years 2005–08 suggested that all the RSV B strains belonged to the BA genotype [27]. It was found that the BA genotype dominated in Pune, Maharashtra during the years 2009–12 [23]. Consequently, the BA viruses from New Delhi attracted our attention during the years 2007–10 [28] and we continued our work on BA genotype throughout the years 2011–15. Interestingly, BA9 was the dominant lineage of the BA genotype identified in our study. Therefore, the aim of the present study was to gain insight into the local and global evolutionary phylodynamics of the BA9 lineage of the BA genotype over the past 16 years. We focused on the second hypervariable region of the G protein gene for Phylogenetic, Bayesian and Network analyses. Such large scale analysis of the global data is envisaged to be useful for undertaking appropriate control measures including possible vaccine development.

## Materials and methods

### Ethical clearance

This study was approved by the Institutional Ethics Committee, Jamia Millia Islamia, New Delhi, India. The study was carried out in accordance with the World Medical Association of Helsinki. Written informed consent was taken in English/Hindi from the parents/guardians of all the pediatric patients before sample collection.

### Clinical samples

Nasopharyngeal/throat swabs were collected from the children less or equal to 5 years of age with acute respiratory infection (ARI) symptoms as defined by WHO [29]. A total of 500 nasopharyngeal/throat swabs were collected from the symptomatic pediatric patients attending the Out Patient Department (OPD) of Dr. M. A. Ansari Health Centre, Jamia Millia Islamia, New Delhi by the Pediatrician.

### Detection of RSV by real-time PCR

All the samples were tested for RSV by real-time PCR. The RNA was extracted from 350μl of nasopharyngeal/throat swabs using RNeasy Mini Kit (Qiagen GmbH, Hilden, Germany) as per the manufacturer’s instructions. The extracted RNA was used for real-time PCR for detection and RT-PCR for characterization. The real-time PCR for the large (L) polymerase gene was carried out using one step real time polymerase chain reaction by SYBR® green chemistry using published primers and cycling conditions [30]. The PCR employed the following thermal cycler conditions: 30 min at 50°C, 15 min at 95°C, followed by 50 cycles of 30 sec at 95°C, 30 sec at 55°C and 30 sec at 72°C. This was followed by melt curve stage analysis using the conditions: 15 sec at 95°C, 1 min at 60°C and 15 sec at 95°C.

### RT-PCR for the 2^nd^ HVR of G protein gene

The cDNA was synthesized from the extracted RNA using High capacity cDNA Reverse Transcription Kits (Applied Biosystem), according to the manufacturer’s protocol. The reaction was performed in a 10μl volume containing 5μl template RNA and 5μl 2X RT master mixture. The reaction was carried out in four steps: 10 min at 25°C, 120 min at 37°C, and 5 min at 85°C followed by incubation at 4°C. The cDNA was stored at -20°C and was used for RT-PCR for the 2^nd^ HVR of G protein gene. Positive samples were subjected to RT-PCR for sub grouping. External and semi nested PCR was done for the 2^nd^ HVR of G protein gene of RSVB using published primers and cycling conditions [20, 26]. The amplicons size of external PCR was 607bp and 670bp for group B and BA genotype. The nested PCR amplicons were of 585/645bp for group B and BA genotype, respectively. The PCR products were run on 2% agarose gel and were visualized with Dolphin-Doc Plus Gel Image system (Wealtec corp, UK).

### DNA sequencing and alignment

The PCR products were extracted from the agarose gel and purified with the Nucleo-pore Sure Extract PCR Clean-up/Gel Extraction Kit (Genetix Biotech Asia Pvt. Ltd New Delhi, India), as per manufacturer’s instructions. The amplicons were sequenced commercially (Applied Bio systems Inc., Foster City, CA, USA) using the published primer BG517 as the forward primer and F164 as the reverse primer [26]. Sequence chromatograms were analyzed with BioEdit version 7.0.9.0 [31] and manually edited in GeneDoc software version 2.7 [32] to resolve nucleotide ambiguities. The RSV sequences were further confirmed by BLAST (http://www.ncbi.nlm.nih.gov/BLAST/).

### DNA sequencing and GenBank accession numbers

Partial nucleotide sequences of the 2^nd^ HVR of the G protein gene of 31 study group B RSV were determined. The sequences were submitted to GenBank (http://www.ncbi.nlm.nih.gov/GenBank/index.html) with the following accession numbers KY078406-KY078435 and KY649190.

### Phylogenetic analysis

Nucleotide sequence of the 2^nd^ HVR of the G protein gene was used for phylogenetic analysis. A multiple sequence alignment was performed for all the available group B RSV sequences with the help of the CLUSTAL W algorithm embedded in BioEdit software. A total of 418 sequences of group B RSV (including 96 strains from India with 31 studied sequences) were used for the phylogenetic analysis (S1 Table). Phylogenetic trees were constructed using the Neighbor-Joining method implemented in the software MEGA 6.06 [33]. Robustness of tree topology was accessed with 1,000 replicates and bootstrap values greater than 70% are shown on the branches of the consensus trees. The nucleotide and amino acid distances were calculated using the Kimura-2 parameter method [34].

### Mutational analysis

The deduced amino acid sequences and the mutations in the study sequence (n = 28) were predicted by comparing with the prototype strain of the BA genotype that was reported from Argentina [19] (accession number: AY333364).

### Glycosylation analysis

N-linked and O-linked amino acid glycosylation in the 2^nd^ HVR of G protein gene of study sequences (n = 28) was predicted by the NetNGlyc 1.0 (http://www.cbs.dtu.dk/services/NetNGlyc/) server and the NetOGlyc 4.0 server (http://www.cbs.dtu.dk/services/NetOGlyc/) respectively (S1 Table).The potential N-linked glycosylation sites were defined as Asn-Xaa-Ser/Thr (where Xaa is not proline). Further, serine/threonine was the potential O-linked sugar acceptors.

### Entropy analysis

The variation in amino acid positions of the 2^nd^ HVR of G protein gene of group B RSV in the alignment dataset was analyzed by Shannon entropy plot implemented in BioEdit [35]. A total of 281 sequences of BA9 lineage (including 28 BA9 studied sequences) were used for this analysis (S1 Table). The threshold value for the Shannon entropy analysis was set to 0.2. The amino acid with entropy value of <0.2 and >0.2 were considering as a conserved and variable amino acids respectively as described earlier [25].

### Selection pressure analysis

The selection pressure in the 2^nd^ HVR of G protein gene of the BA9 lineage of the BA genotype was estimated by using the Datamonkey webserver [36] (http://www.datamonkey.org/). The dataset consisted of 281 sequences from different geographical regions including 28 BA9 strains sequenced in this study (S1 Table). The synonymous (dS), non-synonymous (dN) mutations, positively and negatively selected sites at every codon position were determined using the HKY85, F81, Trn93 and REV nucleotide substitution models by four different algorithms, Single Likelihood Ancestor Counting (SLAC), Fixed Effect Likelihood (FEL) and Internal Fixed Effects Likelihood (IFEL). The selection of sites under positive (dN > dS), neutral (dN = dS) and negative (dN < dS) were determined by cut-off p-value ≤0.2. If an amino acid site met cut-off criteria by at least two methods then it was considered as a positive site. The selection of positive sites was also calculated using the FUBAR algorithm [37]. To consider a positive site, posterior probability (β>α) ≥0.9 was taken for the FUBAR method.

### Phylodynamic analysis

The Markov Chain Monte Carlo (MCMC) method implemented in the software BEAST v1.8.3 [38] was used to compute the time-scaled evolutionary relationship of group B RSV with a particular focus on the BA9 lineage. The study strains and the GenBank sequences from different geographical regions were utilized for the analysis. A total of 307 unique sequences (including study sequences) of group B RSV focusing on the BA9 lineage were used (S1 Table). PAUP 4.0 was used for inferring the phylogenetic tree [39] and Modeltest3.7 program to estimate the nucleotide substitution rate [40]. The best model, favoured by Bay’s factor was calculated by path sampling and stepping stone sampling method [41, 42]. Three clock models (strict, uncorrelated exponential, and uncorrelated lognormal) and four demographic model (constant size, exponential growth, expansion growth, and Bayesian skyline) were used for best fit clock model and demographic model respectively.

All the programs were run on a server provide by CDAC (http://www.cdac.in). The GTR + Invariant + gamma substitution model was selected by Modeltest3.7. Strict clock and exponential growth coalescent tree priors were used to calculate the tMRCA. The program BEAUti v1.8.3 was used for generating the xml file from the nexus file for further use in BEASTv1.8.3. MCMC chain was run for 200 million steps and sampled every 20,000 steps to achieve convergence, which was confirmed with effective sample size (ESS) using Tracer v1.6.0 (http://tree.bio.ed.ac.uk/software/tracer/). Only parameters with an ESS value ≥ 200 were accepted after 10% burn-in [41, 43]. Tree Annotator v1.8.3 was used to construct maximum clade credibility (MCC) trees with the maximum product of posterior probabilities, after removing the first 10% of trees as burn-in. FigTree v1.4.2 (http://tree.bio.ed.ac.uk/software/figtree/) program was used for visualization of the phylogenetic tree with lineages, divergence time scale and nucleotide substitution rate.

### Bayesian skyline plot (BSP) analysis

The demographic changes of a distinct genotype from a particular geographical region were estimated by Bayesian skyline plot analysis in a single population size [44] using nucleotide sequence of the 2^nd^ HVR of G protein gene. Bayesian skyline plots (BSPs) were generated to understand the impression of effective population size of Indian BA9 lineage (n = 65) with respect to time [38]. The details of these individual sequences are given in the Supplementary Table (S1 Table). The graphical interpretation illustrated the changes in the median estimation of relative hereditary diversity (Ne τ) of the virus with time (Ne and τ represents effective population size and generation time respectively). The MCMC method was used for this study implemented in the program BEASTv1.8.3. The generalized time reversible (GTR) was used for nucleotide substitution model selected by Modeltest3.7 mutually with strict clock model and Bayesian skyline coalescent model. MCMC chains were run with 100 million generations and sampled at every 10,000 steps. The skyline plot was generated from the MCMC output using Tracer v1.6.

### Median joining network analysis

The evolutionary relationship between the homologous sequences is usually represented by classical phylogenetic tree based on maximum-likelihood analysis in a trouble-free graphical view. During the evolution process all organisms have passed through some hurdles like microevolution, convergent evolution, and recombination [45]. Therefore, as a result new species or genotypes evolved that are interconnected to each other in complex manner. Keeping these events in mind, for better representation, the Network analysis was performed for a dataset of 307 unique sequences from different geographical regions (S1 Table), using median joining algorithm implemented in Network v 5.0 software [46] (http://www.fluxus-engineering.com). The significance of every pairwise correlation was calculated using a Fisher’s exact test and a Bonferroni correction in DnaSP v. 5.10.01 [47]. This analysis investigates the intricate relationship among haplotypes based on single nucleotide polymorphisms (SNPs) with a minor allele frequency ≥2% in each continental region [48].

## Results

### RSV prevalence in New Delhi, India (2011–15)

All the children less than or equal to 5 years of age with acute respiratory tract infection (ARI) symptoms (WHO, 2005) attending the OPD of Dr. M.A. Ansari Health Centre, Jamia Millia Islamia, New Delhi, India were enrolled for the study. The clinical specimens (nasopharyngeal/throat swabs) were collected from the symptomatic patients by the Pediatrician during the 4 year study period from November 2011 to December 2015. Samples were collected for a period of 6 months during winter season from September 2011 to February 2012 and then for next three years in winter, since RSV is a seasonal disease. Demographic and clinical information about the patients were recorded in the proformas. The clinical symptoms of the patients such as fever, cough, sore throat, breathlessness/tachypnea, chills/rigor and nasal discharge were recorded. Further, RSV infection was correlated with the clinical symptoms, gender, age and positive/negative samples using statistical analysis [49]

Ninety three (18.6%) of the 500 samples collected were positive for RSV. Sixty six samples were positive for group B and 27 samples for group A RSV. Twelve samples were sequenced for group A and 31 for group B RSV. We describe the evolutionary dynamics of the group B RSV with a focus on the BA9 lineage in the present report.

### Local transmission dynamics of the BA9 lineage in India

#### a) Indian BA9 lineage and mutational analysis

**Fig 1:**
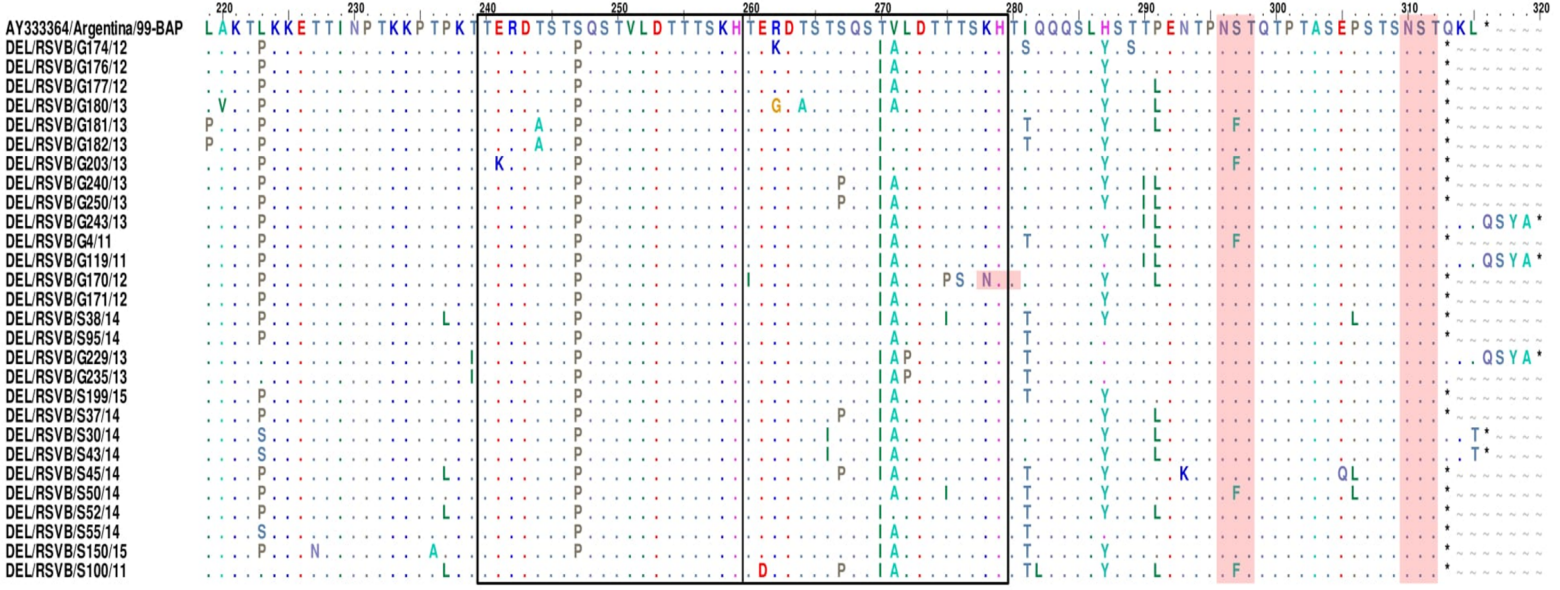
Alignment of deduced amino acid sequences of the 2^nd^ HVR of G protein gene of BA9 lineage with respect to the BA prototype strain (Accession No. AY333364). The alignment corresponds to the G gene (residue positions 219 to 315) of the BA prototype. Identical amino acids are indicated by dots. Asterisks indicate stop codons. Pink shading highlights the predicted potential N-linked glycosylation sites (Asn-Xaa-Ser/Thr sequence context, where Xaa is not a proline).

Phylogenetic analysis of the 31 study sequences revealed that 29 strains clustered in BA genotype (28 BA9 and 1 BA8) and two strains in SAB4 genotype. The prediction of amino acid mutations among the group B study sequences was done in comparison with the BA prototype strain. The BA study strains were predicted to encode a G protein of three different lengths, 312, 315 and 319 amino acids (Fig 1). The G proteins of SAB4 genotype were predicted to be 299 amino acids in length. The genetic distance at the nucleotide and amino acid levels among the BA9 study sequence was 7.4% and 11.7%, respectively. The distance between the study BA sequences and prototype strain at nucleotide and amino acid levels was found to be 0.7% to 7.8% and 1.1% to 11.7%, respectively.

**Fig 2:**
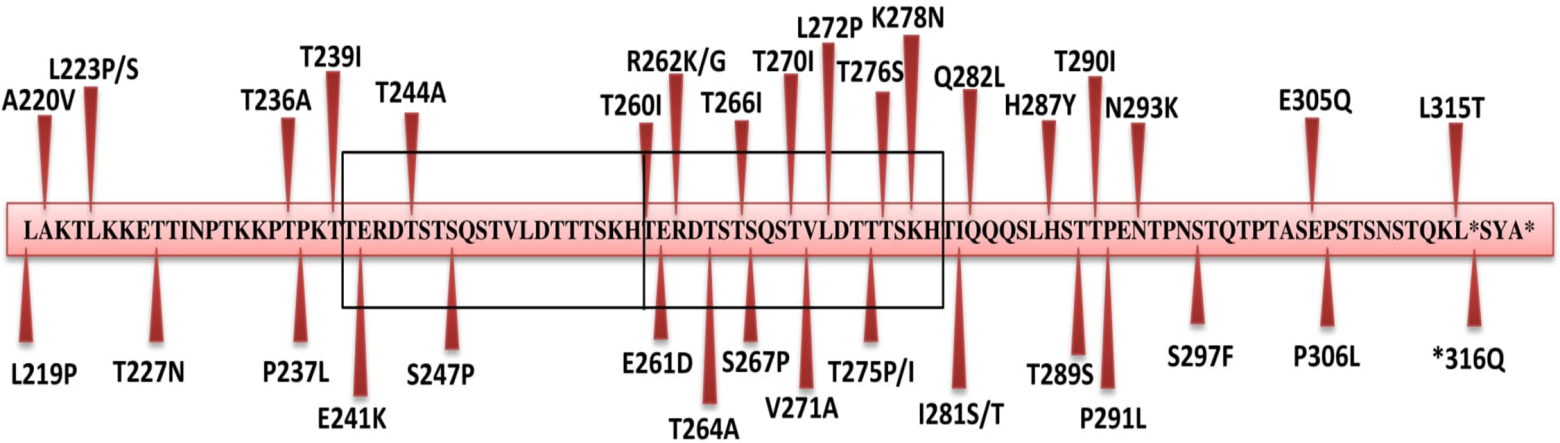
The mutations of deduced amino acid sequence of the 2^nd^ HVR of G gene of BA9 lineage of group B Indian strains including the study sequences. The amino acid residue corresponds to the 219 to 315 of BA prototype strain. Mutations are indicated by arrows.

Substitution mutations were frequently observed in the 2^nd^ HVR of G protein gene of the Indian BA lineage that consisted of 96 sequences. (S1 Fig). A total of 37 amino acid mutations were identified in the studied region (Fig 2).

All the Indian BA9 lineage sequences showed a common substitution mutation at Ser247Pro (except one study sequence DEL/RSVB/S100/11). Further, most of the Indian strains exhibited common changes at four different sites Leu223Pro, Thr270Ile, Val271Ala and His287Tyr that were reported earlier [15]. Substitution mutations within the duplicated region (Ser267Pro, Thr270Ile, and Val271Ala) and in the flanking region (Leu223Pro, Ser247Pro, Ile281Thr and His287Tyr) have been reported earlier [15, 50]. Fifty percent of the study strains exhibited substitutions at Ile281Thr and Pro291Leu. Further, five study strains showed an amino acid change at position Ser297Phe. Three substitutions (Ser267Leu, Ile281Thr and Ser297Phe) were identified to be specific to the BA9 lineage. Twenty one mutations in the studied BA9 sequences were found to be newly identified ones from India. The study strain DEL/RSVB/S45/14 showed the highest number (11) of amino acids substitutions (Fig 1).

#### b) N- and O-linked glycosylation sites of the study strains

N- and O-linked glycosylation sites were investigated for the studied BA9 sequences (n = 28). The sequences had two N-glycosylation sites at 296 and 310 codon positions that were located ahead of the duplication region (Fig 1). The sites at codon positions 296 and 310 were conserved among all the studied strains and in all the BA9 sequences. The studied strain, DEL/RSVB/G170/12 had one additional N-glycosylation site at position 278 in the duplicated region due to substitution of Lys278Arg. Further, 14 to 27 serine and threonine residues were predicted to be the potential O-linked glycosylated sites for strains sequenced in this study with G score ranging from 0.5 to 0.91. One of the study sequence (DEL/RSVB/G235/13) had the maximum number of 27 potential serine and threonine residues. Based on this *in silico* analysis, 8 amino acids were most likely to be glycosylated having G score more than 0.9. These included six threonine residues at 228, 232, 236, 240, 246 and 257 and two serine residues at 245 and 297 amino acids. Two substitutions in most of the studied strains at positions Ser247Pro (analogous region) and Thr270Ile (duplicated region) contributed to the loss of the glycosylation sites. Additionally, another substitution at position 281 in 15 studied strains led to the gain in the glycosylation site. Six additional O-linked glycosylation sites (260, 265, 267, 269, 275 and 277) were identified in the 20 amino acid duplicated region of the strains sequenced in this study.

#### c) Indian BA9 lineage and Bayesian skyline plot (BSP)

**Fig 3:**
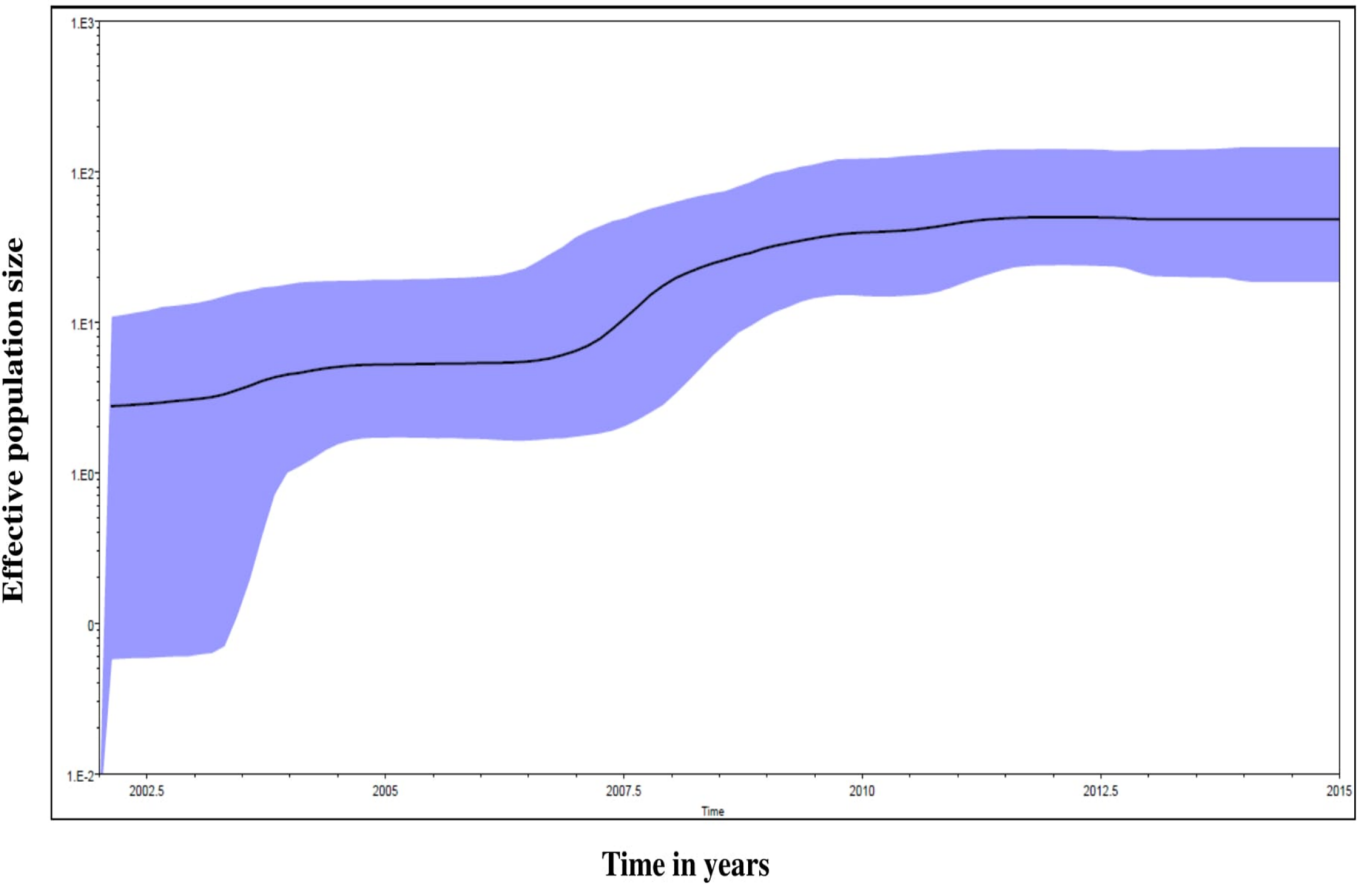
Bayesian skyline plot that describes the demographic history of BA9 lineage of group B RSV in the effective population size with respect to time during the course of evolution in India from 2002 to 2015. The y-axis represents the population size (Net) whereas x-axis indicates the time period in years. The thick lines represent the mean estimate, whereas the transparent area represents the 95% highest posterior density intervals.

BSP analysis was carried out to estimate the effective population size of the Indian BA9 lineage involving time co-ordinates (S1 Table). The results showed significant variation in relative genetic diversity in BA9 lineage including contraction and expansion throughout 14 years of duration from 2002–15 (Fig 3). The effective population of Indian BA9 lineage remained constant until 2007. The population size increased gradually until 2009 and then a constant phase was maintained from 2010 to 2015.

### BA9 lineage and global entropy

**Fig 4:**
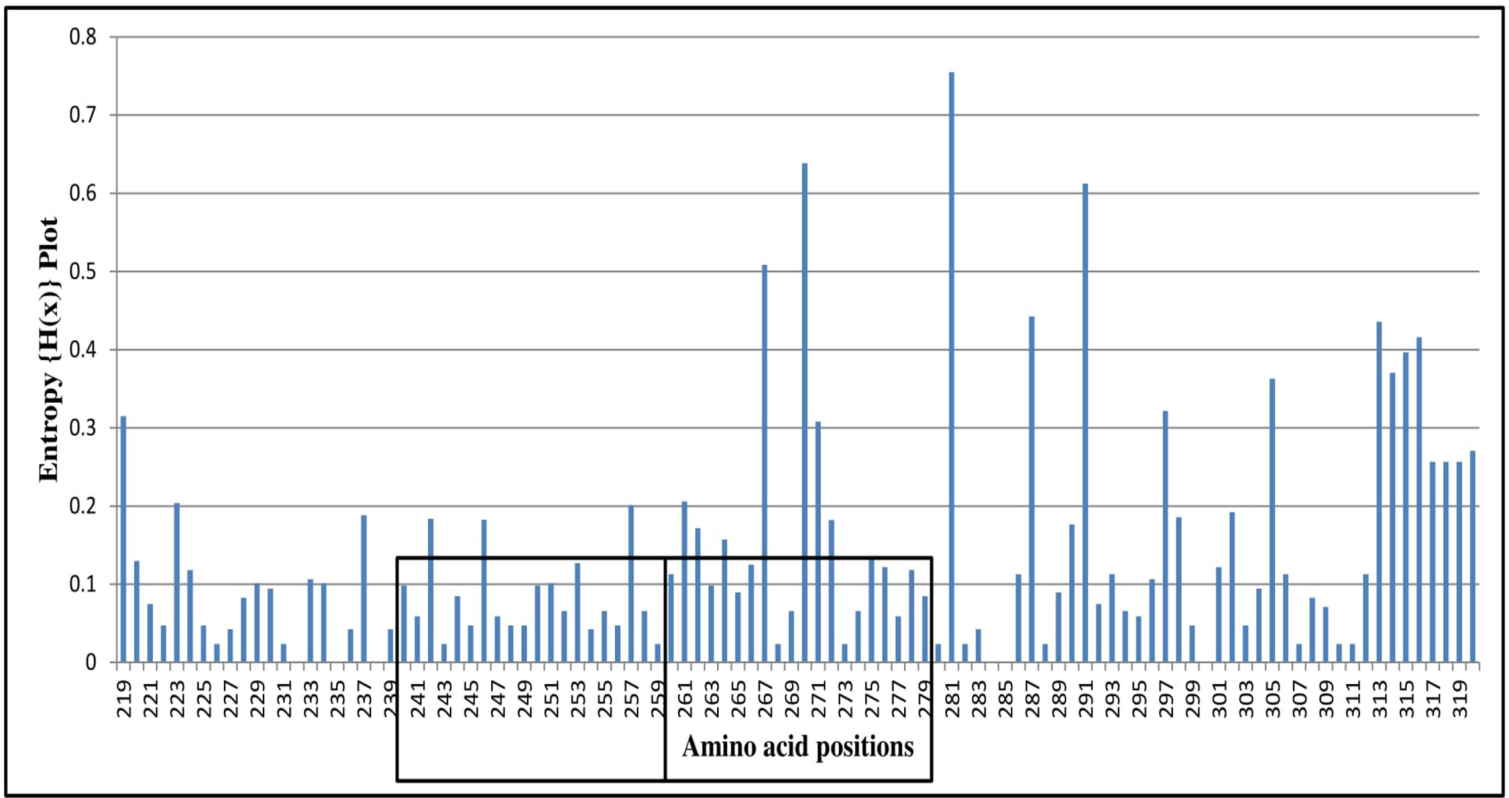
Global amino acid variability of the 2^nd^ HVR of G gene of BA9 lineage is represented by Shannon entropy plot. BioEdit software was used for calculation of entropy values of every amino acid at a particular position. Entropy values <0.2 were considered conserved whereas amino acids with >0.2 values are considered variable. High entropy value showed maximum variability at that particular position.

This analysis revealed that the range of entropy value of the BA9 lineage was 0.00–0.75 with a threshold value of 0.2. A total of 17 variable amino acid sites (219, 267, 270, 271, 281, 287, 291, 297, 305, 313, 314, 315, 316, 317, 318, 319 and 320) were predicted in the BA9 lineage (Fig 4). One of the variable amino acid was identified at position 219 that was present prior to the analogous region. Three sites at positions 267, 270 and 271 were present in the 20 amino acid duplicated region. Thirteen variable sites (281, 287, 291, 297, 305, 313, 314, 315, 316, 317, 318, 319 and 320) were present in the C-terminal region of the G protein gene. One amino acid at position 270 in the duplicated region and two amino acids at positions 281 and 291 had entropy value greater than 0.60. Six amino acid sites (219, 267, 270, 287, 297 and 305) were identified to be positively selected and had high entropy value reflecting variation at these positions. The Indian BA9 lineage showed 21 variable amino acid sites in the analyzed gene of which 5 sites (261, 262, 267, 270 and 271) were present in the duplicated region.

### BA9 lineage and global selection pressure

This analysis revealed a low value of mean dN/dS ratio (0.553–0.630) with a mean value of 0.553 by SLAC method suggesting purifying selection. We found that there were 13 and 7 amino acid sites under positive selection with normalized dN-dS. Thirteen positive sites were confirmed with two different methods: 219, 228, 241, 247, 264, 267, 270, 274, 276, 287, 297, 298 and 305 (Table 1). However, 7 positive sites (219, 267, 270, 276, 287, 297 and 298) were confirmed by all the three methods (Table 1). The selection pressure survival at that particular amino acid positions was due to recurrent mutations (Table 2). A total of 31 negatively selected sites were detected by IFEL method.

**Table 1:** Positively selected amino acid positions of the BA9 lineage.

MODEL→			SLEC		FEL		IFEL		FUBAR	
Genotypes	S. No.	Amino acid positions	Normalised dn-ds	P values	Normalised dn-ds	P values	Normalised dn-ds	P values	β-α values	Post. Pro. β>α
**NA1**	1	219	2.699	0.000	0.445	0.101	0.975	0.040	----	----
	2	228	1.185	0.198	0.388	0.069	----	----	----	----
	3	241	2.348	0.068	0.565	0.109	----	----	----	----
	4	247	1.193	0.195	0.342	0.115	----	----	----	----
	5	264	1.777	0.143	0.656	0.060	----	----	0.238	0.908
	6	267	4.918	0.027	0.814	0.113	1.622	0.046		
	7	270	4.432	0.013	1.024	0.002	0.837	0.049	0.216	0.996
	8	274	----	----	0.291	0.196	0.417	0.188	----	----
	9	276	1.485	0.131	0.492	0.093	0.411	0.191	----	----
	10	287	4.851	0.015	1.163	0.095	1.090	0.175	0.551	0.976
	11	297	2.295	0.050	0.504	0.053	1.154	0.012	----	----
	12	298	1.483	0.132	0.494	0.094	0.843	0.062	----	----
	13	305	----	----	0.238	0.043	0.253	0.101	0.113	0.914

SLAC (single likelihood ancestor counting); FEL (fixed effects likelihood); IFEL (internal fixed effects likelihood); FUBAR (Fast Unconstrained Bayesian AppRoximation)

p-value ≤ 0.2

**Table 2:** Amino acid mutations identified in positively selected sites of the BA9 lineage.

Genotype	Model	Positively selected sites	Mutations observed in positively selection site	Mean dn/ds
**BA9**	**SLAC**	**219, 228, 241, 247, 264, 267, 270, 276, 287, 297, 298**	L219P, L219R, T228I, T228A, R241I, R241G, R241K, R241S, S247P, T264P, T264A, T264I, S267P, S267L, S267A, T270I, T270L, T270F, T270P, T276P, T276A, T276S, H287Y, H287C, H287N, S297F, S297L, S297I, T298A, T298S, T298R	**0.553**
	**FEL**	**219, 228, 241,** **247, 264, 267, 270, 274, 276, 287, 297, 298, 305**	L219P, L219R, T228I, T228A, R241I, R241G, R241K, R241S, S247P, T264P, T264A, T264I, S267P, S267L, S267A, T270I, T270L, T270F, T270P, T274A, T274S, T276P, T276A, T276S, H287Y, H287C, H287N, S297F, S297L, S297I, T298A, T298S, T298R, E305D, E305K, E305Q	**-**
	**IFEL**	**219, 267, 270, 274, 276, 287, 297, 298, 305**	L219P, L219R, S267P, S267L, S267A, T270I, T270L, T270F, T270P, T274A, T274S, T276P, T276A, T276S, H287Y, H287C, H287N, S297F, S297L, S297I, T298A, T298S, T298R, E305D, E305K, E305Q	**-**
	**FUBAR**	**264, 270, 287, 305**	T264P, T264A, T264I, T270I, T270L, T270F, T270P, H287Y, H287C, H287N, E305D, E305K, E305Q	**-**

p-value ≤ 0.2

### Group B RSV and global phylogenetics

**Fig 5:**
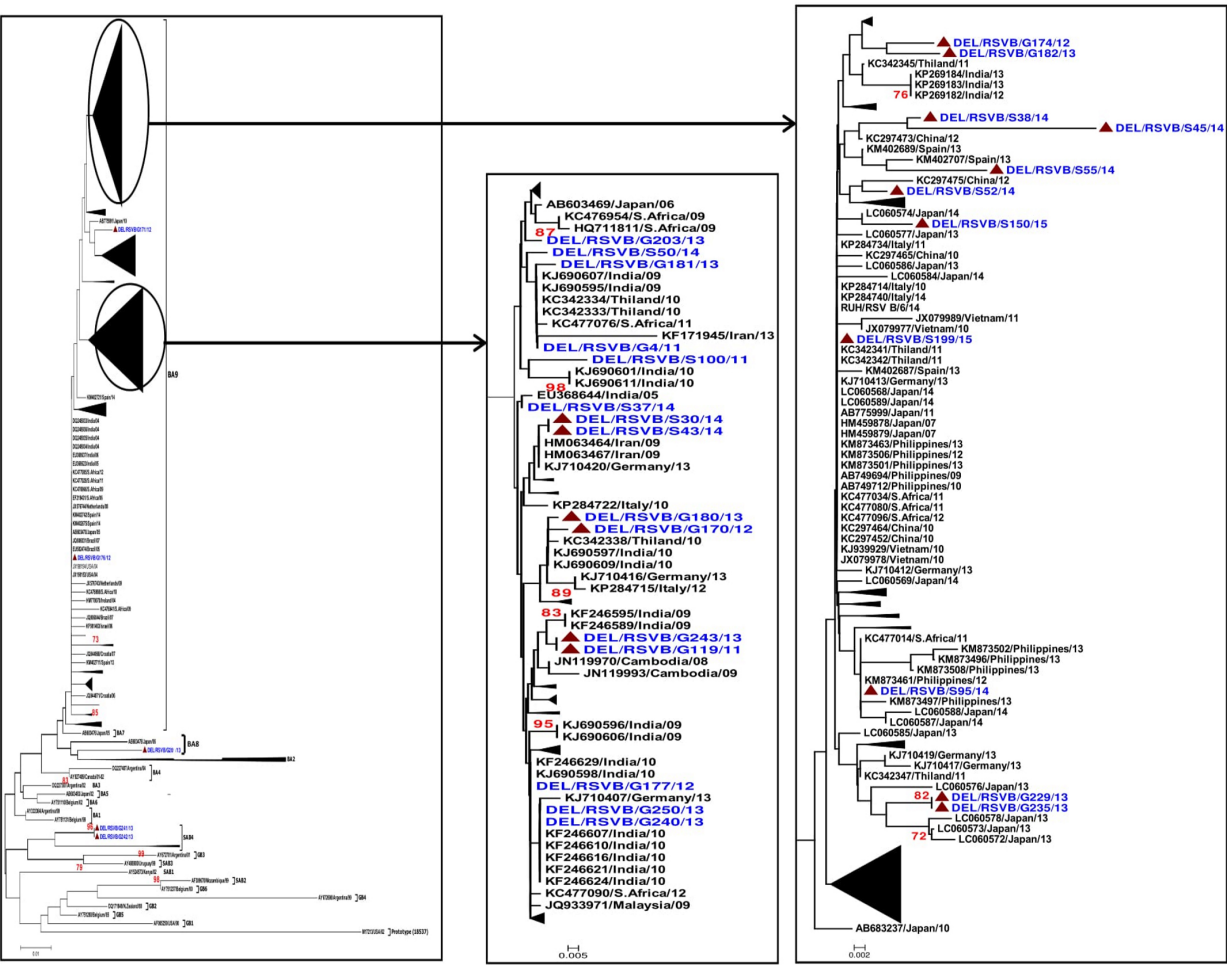
Neighbor-joining phylogenetic tree of the 2^nd^ HVR of G gene of group B RSV; the tree was constructed using Kimura-2 parameter with 1,000 bootstrapping replicates. Only bootstrap values greater than 70% are shown at the branch nodes. The genotypes are indicated at the right by brackets. Prototype strains (M17213/USA/62) were used as an out-group. The study sequences are indicated by solid colored triangles.

A total of 418 sequences of group B RSV (including 31 studied sequences) from 29 different countries were used for the phylogenetic analysis. All the sequences of group B RSV that were available in the GenBank during the period of 1962 to 2015 were used. The region of the alignment spanned from nucleotide 655 to 945 (amino acids 219 to 315) corresponding to the prototype strain of the BA genotype. The analysis revealed that 29 study sequences (93.5%) clustered in the BA genotype. Further, 2 study sequences belonged to the SAB4 genotype (Figs 5 and S2).

### Global distribution and phylodynamics of the BA9 lineage

**Fig 6:**
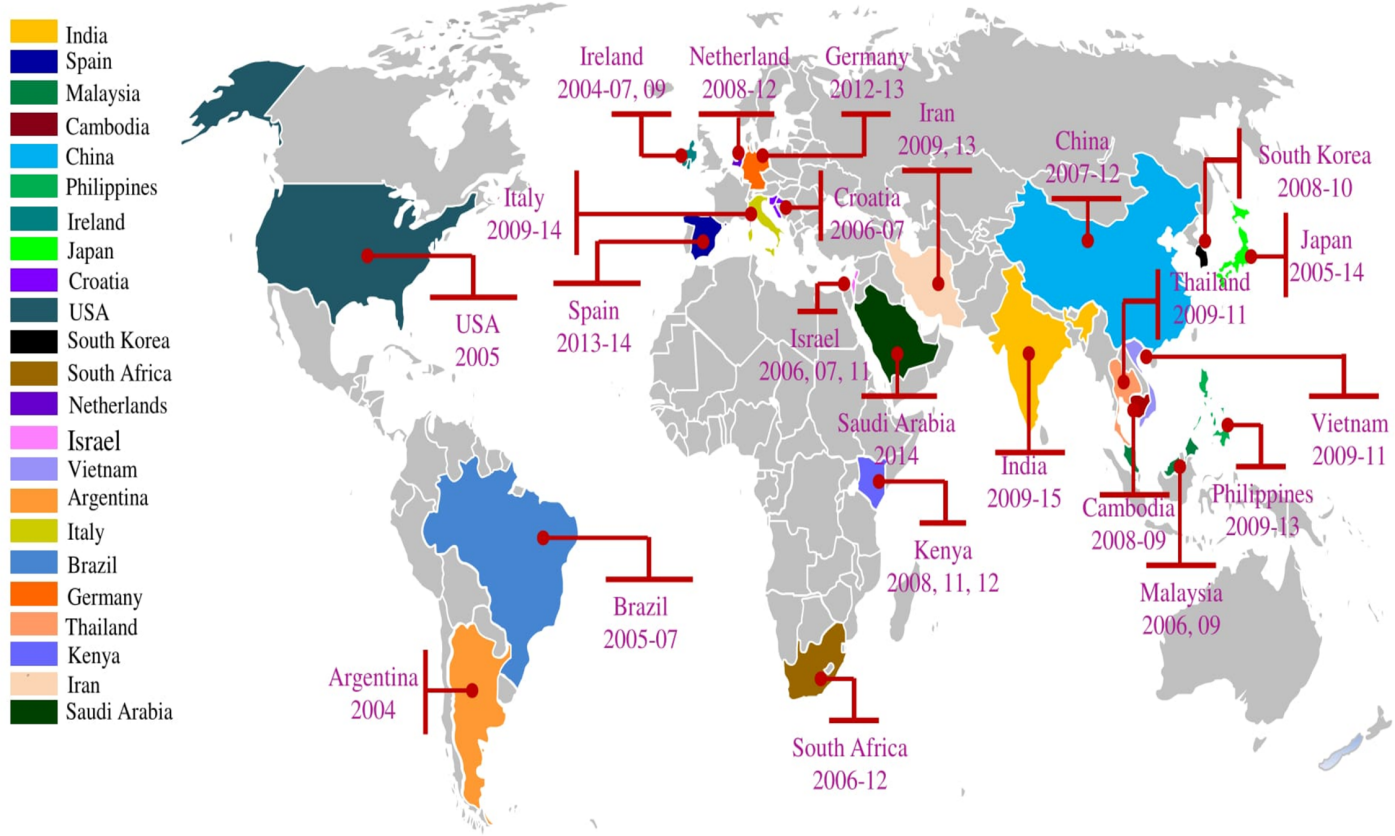
Graphical representation of worldwide distribution of BA9 lineage of group B RSV; Color code bar in left side of the figure is used to represent the countries. The free available editable world map was downloaded from http://www.powerpointslides.net (http://www.powerpointslides.net/powerpointgraphics/powerpointmaps.html). The map was created with PowerPoint and Adobe Photoshop.

The geographical distribution of the BA9 lineage across the globe is depicted in Fig 6. Circulation of the BA9 lineages has been reported from 23 countries including India, Israel, Vietnam, South Korea, Cambodia, China, South Africa, Malaysia, Philippines, Argentina, Netherlands, Spain, Japan, Germany, Thailand, Kenya, Brazil, Croatia, Ireland, Iran, USA, Saudi Arabia, and Italy.

**Fig 7:**
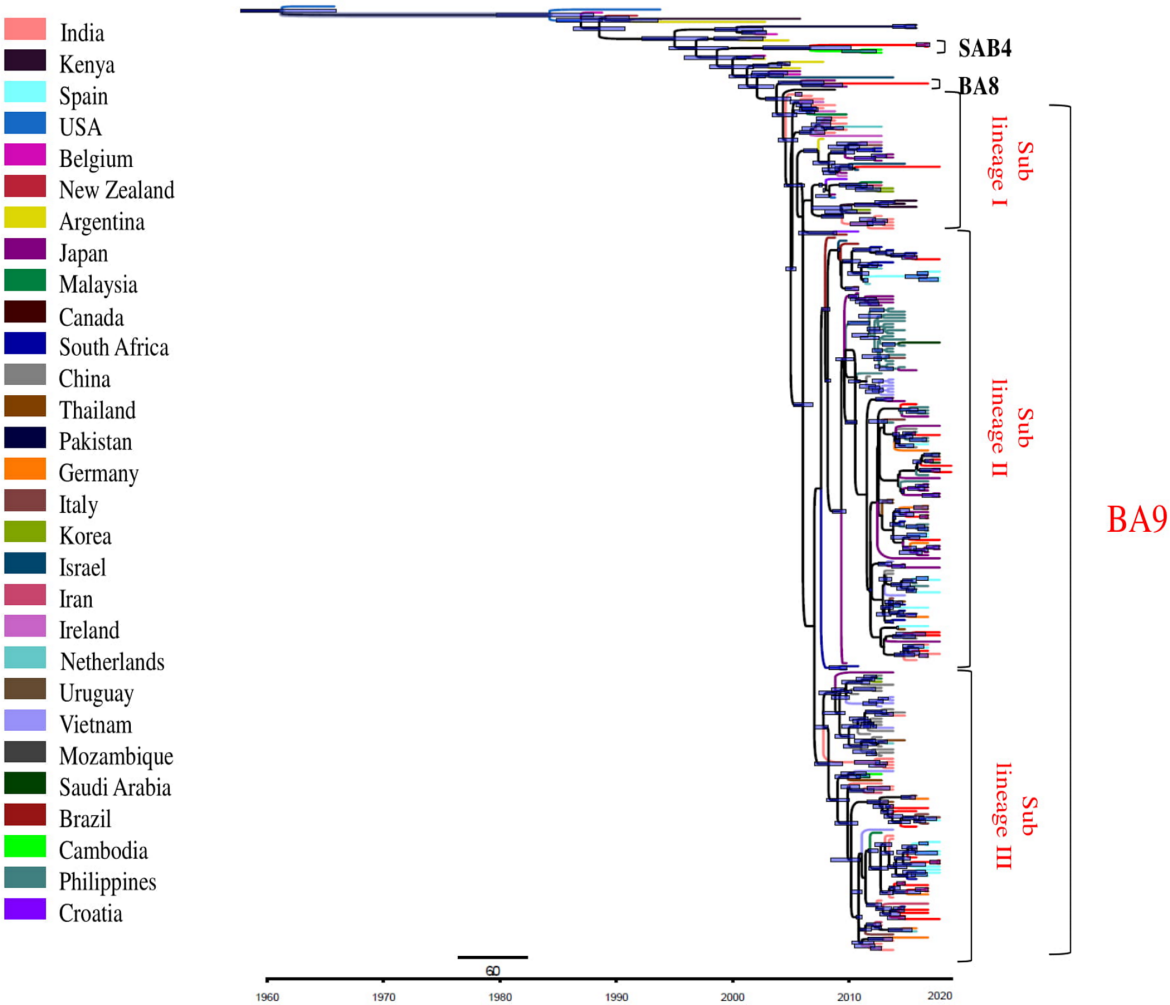
Bayesian MCMC tree of the 2^nd^ HVR of G gene of group B RSV; Nucleotide substitution model (GTR + Invariant + gamma) and exponential growth coalescent tree priors under strict clock was used. Scale bars represent time in years. Gray bars indicate the 95% HPD for the estimated year of divergence. The study sequences are indicated by red colored line. Sequences from different countries in the tree are indicated by colored line at left side of the figure.

Markov Chain Monte Carlo (MCMC) algorithm was used to compute the time-scaled evolutionary relationship to estimate the mean evolutionary and substitution rates of the group B, BA genotype and BA9 lineage (Figs 7 and S3). The analysis revealed that the group B, BA genotype and BA9 lineage deviated from their ancestors during the years 1955 (95% HPD; 1947–1962), 1995 (95% HPD; 1987–1997) and 2000 (95% HPD; 1998–2001), respectively (Table 3). The global mean evolutionary rates (substitution/site/year) were determined for the group B RSV, BA genotype and BA9 lineage. The evolutionary rate of group B RSV was 4.59×10^−3^ (95% HPD; 3.88–5.30×10^−3^) that was faster than that for group A RSV i.e 3.49×10^−3^ (95% HPD, 2.90–4.17×10^−3^). The evolutionary rate of the BA genotype was 4.58×10^−3^ (3.89–5.29×10^−3^) and that of the BA9 lineage was 4.03×10^−3^ (95% HPD; 4.65–5.25×10^−3^) (Table 3).

**Table 3:** Estimated mean evolutionary rate and tMRCA of group B RSV.

Genotypes	Mean evolutionary rate (95% HPD) (substitution/site/year)	Mean tMRC (95% HPD)
All RSVB	4.59 × 10^−3^ (3.88 × 10^−3^–5.30 × 10^−3^)	1955 (1947–1962)
BA	4.58 × 10^−3^ (3.89 × 10^−3^–5.29 × 10^−3^)	1995 (1987–1997)
BA9	4.03 × 10^−3^ (4.65 × 10^−3^–5.25 × 10^−3^)	2000 (1998–2001)

The BA9 lineage was categorized into 3 sub-lineages (I, II and III) within about 16 years of its origin (Figs 7 and S3). Sub-lineage I (48 sequences) was reported from different continents including Asia (India, Malaysia, Japan, South Korea, Israel and Iran), Europe (Ireland, Netherland, Italy and Croatia), Africa (South Africa and Kenya) and Americas (USA, Argentina and Brazil). Eleven sequences from India clustered within this sub-lineage. Further, sub-lineage II (141 sequences) was described from Asia (India, Japan, Philippines, China, Vietnam, Thailand, Israel and Saudi Arabia), Europe (Netherlands, Spain, Ireland, Italy and Germany) Africa (South Africa) and Americas (Brazil). This sub-lineage included 22 sequences from India. Similarly sub-lineage III (91 sequences) included sequences from Asia (India, Japan, China, Malaysia, South Korea, Vietnam, Thailand, Iran and Cambodia), Europe (Netherlands, Italy, Germany and Spain) and Africa (South Africa). Thirty one sequences from India clustered in this sub-lineage. Sequences from the following countries India, Italy, Japan, Netherland, and South Africa clustered in all the sub-lineages. The studied strains clustered in all the sub-lineages I (1 sequence), II (15 sequences) and III (13 sequences).

### BA9 lineage and global phylogeographics

**Fig 8:**
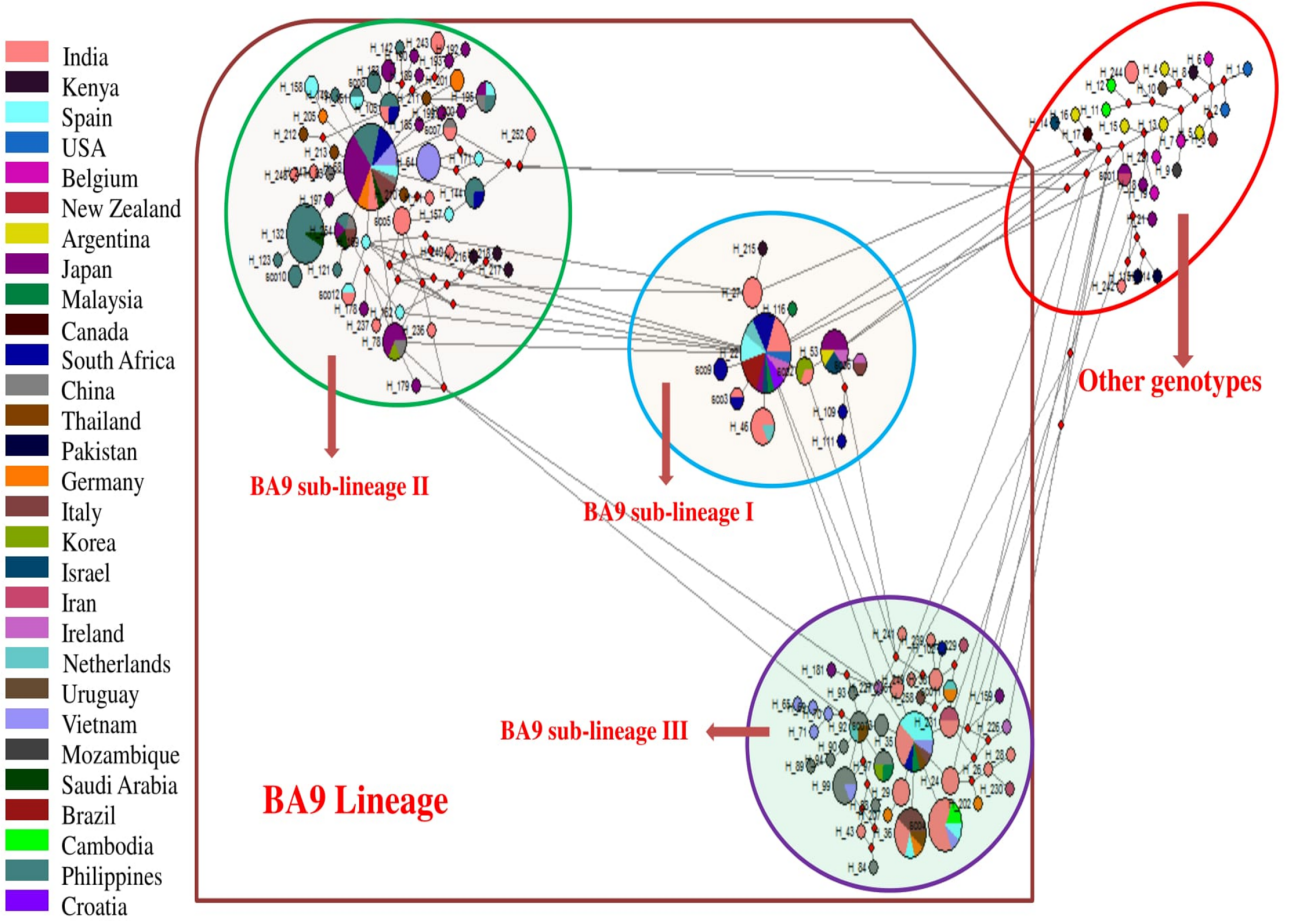
Median joining network of the 2^nd^ HVR of G gene of group B depicting the relationship between the strains or lineages. The length of line between the haplotypes does not depend on the number of mutations. Each circle represents the haplotypes and surface area of each circle reflects the frequency. Haplotypes circulating in different countries were represented by colour-codes. Coloured segments inside the circle indicate the shared haplotypes. Median vectors are indicated by red circles.

The global phylogeographic analysis was carried out for the BA9 lineage. We obtained 190 variable sites that defined 129 haplotypes (H) with 0.9954 diversity (Hd). The frequency of ≥ 2 was used to allow a large number of haplotypes with maximum single nucleotide polymorphisms. The H1 (M17213/USA/62) was progenitor of all the strains of group B RSV, whereas all the lineages of the BA genotype (BA2-12) emerged from the precursor H13 (AY333364/Argentina/99) (Fig 8). All the RSV strains in the network were interconnected and formed 3 sub-lineages, (I-III). The distribution of the sequences in these 3 sub-lineages was similar to the categorization of the 3 sub-lineages described by the Bayesian analysis (Fig 7). There were 25 haplotypes of strains sequenced in this study clustered in all three sub-lineages. The network analysis showed that BA9 lineage evolved from its progenitors BA7 and BA8 lineages.

## Discussion

RSV is an important pathogen of ARI that has global distribution. The BA and ON1 genotypes of RSV have 60 and 72bp duplication in the 2^nd^ HVR of the G protein gene [11, 14]. These two genotypes with duplications spread across the globe within a few years of their identification probably due to the immunologically naive host population [51]. A recent study described the global evolutionary dynamics of the ON1 genotype that has been categorized into 3 lineages (1.1, 1.2 and 1.3) [52]. Similarly, the BA genotype has been divided into 12 lineages; (BA1-12). We aimed to describe the local and global transmission dynamics of group B RSV with focus on the BA9 lineage. We identified RSV strains during molecular surveillance of ARI from New Delhi, India recorded consecutively for 4 years from 2011 to 2015. The global distribution and evolutionary dynamics of the NA1 genotype has been described in a recent report [49]. The local transmission dynamics of RSV in India was investigated owing to paucity of information on its molecular epidemiology. This included 96 BA strains reported from India during the past 14 years from 2002 to 2015. Significantly, BA viruses have either partly or fully replaced the previously circulating non-BA genotypes across different geographical regions [8, 10, 17, 18, 23, 24, 51, 53–59]. However, some of the non-BA genotypes continued to circulate globally at moderate levels. An examination of the Bayesian skyline plot of the Indian BA9 lineage suggested contraction and expansion of the total virus population size during the years 2002–15. Further, surveillance will determine the epidemiological characteristics of this genotype in India for its continued presence or absence altogether.

The genetic variations in the G gene of RSV are caused due to point mutations, large duplications events and use of alternative stop codons. The genetic variations in the 60bp duplicated region in the BA genotype have resulted in formation of different lineages (BA1 to BA12). Further, genetic variations among these lineages may form sub-lineages. Similarly, the antigenic variations occur due to alterations of the N- and O-linked glycosylation sites. Although several amino acid mutations were identified in the BA9 lineage, 12 of these substitutions were mapped exclusively to the 60bp duplicated region suggesting evolutionary selection pressure on this part of the gene [60, 61]. Four of these mutations showed positive selection whereas two substitutions had high entropy values. The amino acid at positions 267 and 270 in the duplicated region had high entropy and showed positive selection as described below. The global selection pressure analysis of the BA9 lineage revealed low mean dN/dS ratio suggesting that codon positions are conserved. Further, 13 positively selected sites were identified in the BA9 lineage suggesting its stochastic route of evolution [61]. Interestingly, six different amino acids (219, 267, 270, 287, 297 and 305) were positively selected and had high entropy values indicating variations at these positions. The amino acid at positions 267 and 270 were reported to have high entropy value from Philippines and Saudi Arabia and 219 from Saudi Arabia alone [25, 51]. In addition, 4 positively selected amino acids (positions 228, 267, 297 and 298) identified in the studied strains also showed O-linked glycosylation. Further, six additional O-linked glycosylation sites were identified in the 20 amino acid duplicated region of the BA9 lineage. Taken together, the genetic and antigenic alterations seems to provide additional evolutionary advantages to the BA9 viruses leading to evasion of host immune response that might contribute towards their enhanced transmission and thus global dispersal.

We carried out the global phylogenetic analysis of the group B RSV sequences reported from 29 different countries. The group B sequences clustered in different genotypes including GB1-GB-6, SAB1-SAB4 and BA. BA9 is the most prevalent lineage of the BA viruses that has been described in recent publications [60]. Therefore, we assessed the global evolutionary dynamics of BA9 lineage that was circulating in 23 different countries. The global spread of the BA9 lineage might be associated with travelers [62, 63] or selective fitness advantages. The 60bp duplication in the BA9 lineage might contribute to altering the antigenic characteristics of the virus leading to immune evasion thus providing selective advantage for transmission in the human host. Phylogenetic analysis showed that studied group B strains clustered in the BA (29 sequences) and SAB4 (2 sequences) genotypes. The BA genotype included 28 sequences of the BA9 lineage whereas 1 strain clustered with the BA8 lineage. We analysed the BA9 lineage of BA genotype in detail since it was the predominant lineage. This analysis revealed global distribution of the BA9 genotype that was reflected across different geographical regions including India, Spain, Philippines, Thailand, South Africa, Japan, China, Vietnam, Ireland, Italy, Israel, Argentina, Iran, Germany, Cambodia and Malaysia [8, 10, 13–15, 17, 21, 23, 24, 26, 28, 51, 55, 57, 64–81]. Further, the studied SAB4 strains clustered with sequences from Cambodia [13]. The studied BA8 strain clustered with the sequence from Japan [72]

We estimated the divergent year for the tree root of group B RSV. The analysis revealed that group B, BA genotype and BA9 lineage deviated from their ancestors in the years 1955, 1995 and 2000, respectively. It seems all existing strains of group B RSV originated in 1955 [8, 18, 24, 56, 58, 59]. The mean evolutionary rate was determined for different datasets including group A, group B, BA genotype and BA9 lineage. The rate of group B RSV [4.59×10^−3^ (95% HPD; 3.88–5.30×10^−3^)] was faster than that for group A viruses [3.49×10^−3^ (95% HPD; 2.90–4.17×10^−3^)]. This suggested that group B viruses are probably evolving faster than their group A counterpart as reported earlier [12, 43, 69]. The substitution rate of group B RSV was previously reported from Japan [4.88×10^−3^ (3.97–5.83×10^−3^)] and Italy [3.03×10^−3^ (2.1–3.9×10^−3^)] [24, 58]. However, the BA9 lineage showed marginally different global evolutionary rate of 4.03×10^−3^ (95% HPD; 4.65–5.25 ×10^−3^) as compared to that of BA genotype [4.58×10^−3^ (3.89–5.29×10^−3^)]. The evolutionary rate of the BA9 estimated in the present study was within the 95% HPD reported from Japan [4.77×10^−3^ (3.27–6.39×10^−3^)] [24]. This recent report analyzed the evolutionary dynamics of the BA9 and BA10 lineages with limited GenBank sequences. We described comprehensive global evolutionary trajectory of the BA9 lineage along with its local transmission dynamics. Interestingly, the global evolutionary rate of BA9 (with 60bp duplication) was found to be similar to that of the ON1 genotype (with 72bp duplication) [4.10 ×10^−3^ (95% HPD; 3.1–5.0×10^−3^)] [52]. Taken together, the genotype of group A and lineage of group B that possess duplications (ON1 and BA9 respectively) seem to be evolving at similar rates. This similarity of duplication among ON1 and BA9 may be due to the immunological naive scenario of the human population. We estimated the evolutionary rate of the 2^nd^ HVR of the G gene which tend to accumulate mutations affecting the immunological response. An insight into the evolutionary rates of the full length G gene and genome of BA9 lineage is envisaged to elucidate the detailed evolutionary dynamics of this rapidly evolving respiratory pathogen.

We found that BA9 lineage has emerged in the year 2000 and diverged substantially into 3 global sub-lineages. However, most of the BA9 sequences grouped into the sub-lineage II that showed frequent global spread. All the sub-lineages of BA9 showed somewhat temporal distribution. In this context, the strains described during the year 2002–06 clustered in the sub-lineage I. The recently reported strains from 2013–15 belonged to sub-lineage II and III whereas the strains reported during the year 2007–12 clustered in all the three sub-lineages. Thus, different lineages seem to have followed varying trajectory of evolution to survive efficiently in the human host.

## Conclusions

The analysis of the BA9 lineage showing its global spread with higher evolutionary rate warrants community and hospital based surveillance across different geographical regions. Since the virus has three distinct global lineages, it would be of interest to monitor their individual or collective infection in the human host to uncover their survival strategies. Finally, work undertaken on host immune response in the context of different lineages will uncover the possible molecular mechanism of infection.

## Supporting information

S1 Fig Alignment of the deduced amino acid sequences of 2^nd^ HVR of G protein gene of BA9 lineage of the BA genotype of group B RSV. The alignment includes 96 sequences of the BA9 lineage that have been reported from India.
(PDF)

S2 Fig Neighbor-joining phylogenetic tree of 2^nd^ HVR of G protein gene of group B RSV. (PDF)

S3 Fig MCC tree of 2^nd^ HVR of G protein gene of group B RSV. (PDF)

S1 Table Details of the sequences used for the phylogenetic, Bayesian, Network, skyline plot, selection pressure, entropy and glycosylation analyses. (DOCX)
