# Supplementary material for: BA9 lineage of respiratory syncytial virus from across the globe and its evolutionary dynamics
Source: PLoS One. 2018 Apr 25;13(4):e0193525. doi: 10.1371/journal.pone.0193525 (PMC5919079; doi:10.1371/journal.pone.0193525)
Supplement: S3 Fig — (PDF) [file pone.0193525.s003.pdf]

BA9 lineage of Respiratory Syncytial Virus from across the Globe and its Evolutionary Dynamics

Md Shakir Hussain Haider, Wajihul Hasan Khan, Farah Deeba, Sher Ali, Anwar Ahmed, Irshad H Naqvi, Ravins Dohare, Hytham A. Alsenaidy, Abdulrahman M. Alsenaidy, Shobha Broor, Shama Parveen

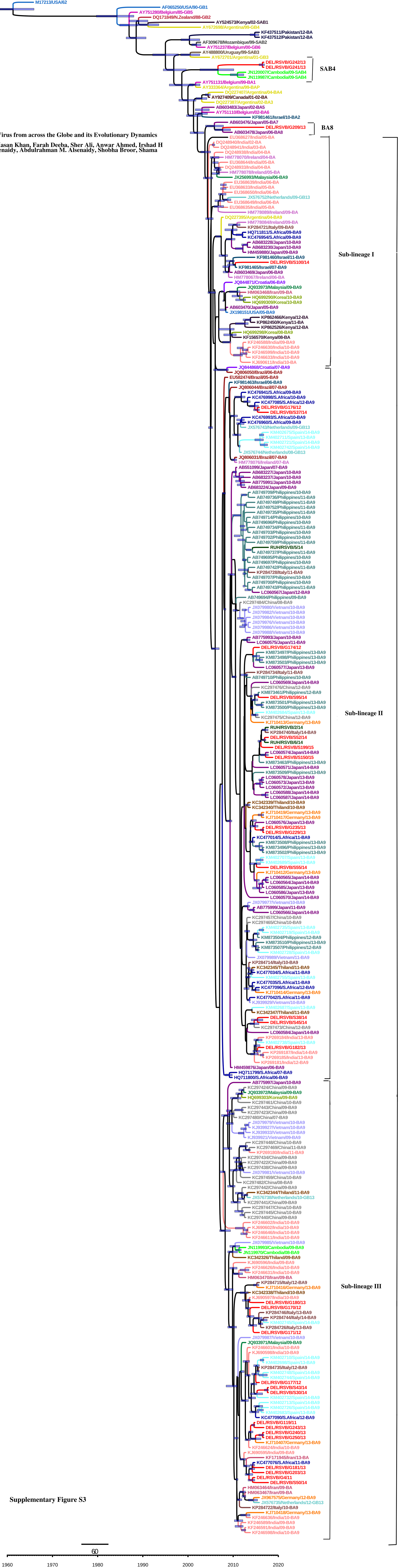

Supplementary Figure S3
