# Supplementary material for: BA9 lineage of respiratory syncytial virus from across the globe and its evolutionary dynamics
Source: PLoS One. 2018 Apr 25;13(4):e0193525. doi: 10.1371/journal.pone.0193525 (PMC5919079; doi:10.1371/journal.pone.0193525)
Supplement: S1 Table — (DOCX) [file pone.0193525.s004.docx]

**Supplementary Table 1. Details of the sequences used for the phylogenetic, Bayesian, Network, skyline plot, selection pressure, entropy and glycosylation analyses**

| **GenBank Accession No.** | **Country/ Year of isolation** | **Genotype** | **Phylogenetic analysis** | **BEAST analysis** | **BSP analysis** | **Network** | **Selection pressure analysis/ Entropy analysis** | **N-and O- linked glycosylation** |
| --- | --- | --- | --- | --- | --- | --- | --- | --- |
| KY078406 | India/12 | BA9 | ✓ | ✓ | ✓ | ✓ | ✓ | ✓ |
| KY078407 | India/12 | BA9 | ✓ | ✓ | ✓ | ✓ | ✓ | ✓ |
| KY078408 | India/13 | BA9 | ✓ | ✓ | ✓ | ✓ | ✓ | ✓ |
| KY078409 | India/13 | BA9 | ✓ | ✓ | ✓ | ✓ | ✓ | ✓ |
| KY078410 | India/13 | BA9 | ✓ | ✓ | ✓ | ✓ | ✓ | ✓ |
| KY078411 | India/13 | BA9 | ✓ | ✓ | ✓ | ✓ | ✓ | ✓ |
| KY078412 | India/13 | BA9 | ✓ | ✓ | ✓ | ✓ | ✓ | ✓ |
| KY078413 | India/13 | BA9 | ✓ | ✓ | ✓ | ✓ | ✓ | ✓ |
| KY078414 | India/13 | BA9 | ✓ | ✓ | ✓ | ✓ | ✓ | ✓ |
| KY078415 | India/11 | BA9 | ✓ | ✓ | ✓ | ✓ | ✓ | ✓ |
| KY078416 | India/11 | BA9 | ✓ | ✓ | ✓ | ✓ | ✓ | ✓ |
| KY078417 | India/12 | BA9 | ✓ | ✓ | ✓ | ✓ | ✓ | ✓ |
| KY078418 | India/12 | BA9 | ✓ | ✓ | ✓ | ✓ | ✓ | ✓ |
| KY078419 | India/13 | BA8 | ✓ | ✓ | 🗶 | ✓ | 🗶 | 🗶 |
| KY078420 | India/14 | BA9 | ✓ | ✓ | ✓ | ✓ | ✓ | ✓ |
| KY078421 | India/14 | BA9 | ✓ | ✓ | ✓ | ✓ | ✓ | ✓ |
| KY078422 | India/13 | BA9 | ✓ | ✓ | ✓ | ✓ | ✓ | ✓ |
| KY078423 | India/13 | BA9 | ✓ | ✓ | ✓ | ✓ | ✓ | ✓ |
| KY078424 | India/15 | BA9 | ✓ | ✓ | ✓ | ✓ | ✓ | ✓ |
| KY078425 | India/14 | BA9 | ✓ | ✓ | ✓ | ✓ | ✓ | ✓ |
| KY078426 | India/14 | BA9 | ✓ | ✓ | ✓ | ✓ | ✓ | ✓ |
| KY078427 | India/14 | BA9 | ✓ | ✓ | ✓ | ✓ | ✓ | ✓ |
| KY078428 | India/14 | BA9 | ✓ | ✓ | ✓ | ✓ | ✓ | ✓ |
| KY078429 | India/14 | BA9 | ✓ | ✓ | ✓ | ✓ | ✓ | ✓ |
| KY078430 | India/14 | BA9 | ✓ | ✓ | ✓ | ✓ | ✓ | ✓ |
| KY078431 | India/15 | BA9 | ✓ | ✓ | ✓ | ✓ | ✓ | ✓ |
| KY078432 | India/11 | BA9 | ✓ | ✓ | ✓ | ✓ | ✓ | ✓ |
| KY078433 | India/12 | BA9 | ✓ | ✓ | ✓ | ✓ | ✓ | ✓ |
| KY078434 | India/13 | SAB4 | ✓ | ✓ | 🗶 | ✓ | 🗶 | 🗶 |
| KY078435 | India/13 | SAB4 | ✓ | ✓ | 🗶 | ✓ | 🗶 | 🗶 |
| KY649190 | India/14 | BA9 | ✓ | ✓ | ✓ | ✓ | ✓ | ✓ |
| DQ248937 | India/04 | BA | ✓ | 🗶 | 🗶 | 🗶 | 🗶 | 🗶 |
| DQ248933 | India/04 | BA | ✓ | ✓ | ✓ | ✓ | ✓ | 🗶 |
| KF246646 | India/10 | BA9 | ✓ | ✓ | ✓ | ✓ | ✓ | 🗶 |
| KF246645 | India/10 | BA9 | ✓ | 🗶 | 🗶 | 🗶 | 🗶 | 🗶 |
| KF246636 | India/10 | BA9 | ✓ | ✓ | ✓ | ✓ | ✓ | 🗶 |
| KF246633 | India/10 | BA9 | ✓ | ✓ | ✓ | ✓ | ✓ | 🗶 |
| KF246631 | India/10 | BA9 | ✓ | ✓ | ✓ | ✓ | ✓ | 🗶 |
| KF246630 | India/10 | BA9 | ✓ | ✓ | ✓ | ✓ | ✓ | 🗶 |
| KF246629 | India/10 | BA9 | ✓ | 🗶 | 🗶 | 🗶 | 🗶 | 🗶 |
| KF246627 | India/10 | BA9 | ✓ | 🗶 | 🗶 | 🗶 | 🗶 | 🗶 |
| KF246626 | India/10 | BA9 | ✓ | ✓ | ✓ | ✓ | ✓ | 🗶 |
| KF246624 | India/10 | BA9 | ✓ | ✓ | ✓ | ✓ | ✓ | 🗶 |
| KF246621 | India/10 | BA9 | ✓ | 🗶 | 🗶 | 🗶 | 🗶 | 🗶 |
| KF246616 | India/10 | BA9 | ✓ | 🗶 | 🗶 | 🗶 | 🗶 | 🗶 |
| KF246611 | India/10 | BA9 | ✓ | ✓ | ✓ | ✓ | ✓ | 🗶 |
| KF246610 | India/10 | BA9 | ✓ | 🗶 | 🗶 | 🗶 | 🗶 | 🗶 |
| KF246607 | India/10 | BA9 | ✓ | 🗶 | 🗶 | 🗶 | 🗶 | 🗶 |
| KF246602 | India/10 | BA9 | ✓ | ✓ | ✓ | ✓ | ✓ | 🗶 |
| KF246601 | India/10 | BA9 | ✓ | ✓ | ✓ | ✓ | ✓ | 🗶 |
| KF246599 | India/10 | BA9 | ✓ | ✓ | ✓ | ✓ | ✓ | 🗶 |
| KF246598 | India/10 | BA9 | ✓ | ✓ | ✓ | ✓ | ✓ | 🗶 |
| KF246595 | India/09 | BA9 | ✓ | 🗶 | 🗶 | 🗶 | 🗶 | 🗶 |
| KF246591 | India/09 | BA9 | ✓ | ✓ | ✓ | ✓ | ✓ | 🗶 |
| KF246589 | India/09 | BA9 | ✓ | ✓ | ✓ | ✓ | ✓ | 🗶 |
| KF246588 | India/09 | BA9 | ✓ | ✓ | ✓ | ✓ | ✓ | 🗶 |
| KJ690609 | India/10 | BA | ✓ | 🗶 | 🗶 | 🗶 | 🗶 | 🗶 |
| KJ690607 | India/09 | BA | ✓ | 🗶 | 🗶 | 🗶 | 🗶 | 🗶 |
| KJ690602 | India/10 | BA9 | ✓ | ✓ | ✓ | ✓ | ✓ | 🗶 |
| KJ690601 | India/10 | BA | ✓ | 🗶 | 🗶 | 🗶 | 🗶 | 🗶 |
| KJ690598 | India/10 | BA9 | ✓ | ✓ | ✓ | ✓ | ✓ | 🗶 |
| KJ690597 | India/10 | BA9 | ✓ | ✓ | ✓ | ✓ | ✓ | 🗶 |
| KJ690596 | India/09 | BA9 | ✓ | ✓ | ✓ | ✓ | ✓ | 🗶 |
| KJ690595 | India/09 | BA9 | ✓ | ✓ | ✓ | ✓ | ✓ | 🗶 |
| KJ690594 | India/10 | BA9 | ✓ | 🗶 | 🗶 | 🗶 | 🗶 | 🗶 |
| KJ690611 | India/10 | BA | ✓ | ✓ | ✓ | ✓ | ✓ | 🗶 |
| KJ690608 | India/10 | BA | ✓ | 🗶 | 🗶 | 🗶 | 🗶 | 🗶 |
| KJ690606 | India/09 | BA | ✓ | 🗶 | 🗶 | 🗶 | 🗶 | 🗶 |
| KP269187 | India/14 | BA9 | ✓ | ✓ | ✓ | ✓ | ✓ | 🗶 |
| KP269186 | India/13 | BA9 | ✓ | 🗶 | 🗶 | 🗶 | 🗶 | 🗶 |
| KP269185 | India/13 | BA9 | ✓ | ✓ | ✓ | ✓ | ✓ | 🗶 |
| KP269184 | India/13 | BA9 | ✓ | ✓ | ✓ | ✓ | ✓ | 🗶 |
| KP269183 | India/13 | BA9 | ✓ | 🗶 | 🗶 | 🗶 | 🗶 | 🗶 |
| KP269182 | India/12 | BA9 | ✓ | 🗶 | 🗶 | 🗶 | 🗶 | 🗶 |
| KP269181 | India/12 | BA9 | ✓ | ✓ | ✓ | ✓ | ✓ | 🗶 |
| KP269180 | India/11 | BA9 | ✓ | ✓ | ✓ | ✓ | ✓ | 🗶 |
| DQ248941 | India/03 | BA | ✓ | ✓ | ✓ | ✓ | ✓ | 🗶 |
| DQ248940 | India/02 | BA | ✓ | ✓ | ✓ | ✓ | ✓ | 🗶 |
| DQ248938 | India/04 | BA | ✓ | ✓ | ✓ | ✓ | ✓ | 🗶 |
| DQ248936 | India/04 | BA | ✓ | 🗶 | 🗶 | 🗶 | 🗶 | 🗶 |
| DQ248935 | India/04 | BA | ✓ | 🗶 | 🗶 | 🗶 | 🗶 | 🗶 |
| DQ248934 | India/04 | BA | ✓ | 🗶 | 🗶 | 🗶 | 🗶 | 🗶 |
| EU368650 | India/06 | BA | ✓ | ✓ | ✓ | ✓ | ✓ | 🗶 |
| EU368649 | India/06 | BA | ✓ | ✓ | ✓ | ✓ | ✓ | 🗶 |
| EU368644 | India/05 | BA | ✓ | ✓ | ✓ | ✓ | ✓ | 🗶 |
| EU368642 | India/07 | BA | ✓ | 🗶 | 🗶 | 🗶 | 🗶 | 🗶 |
| EU368640 | India/05 | BA | ✓ | 🗶 | 🗶 | 🗶 | 🗶 | 🗶 |
| EU368639 | India/06 | BA | ✓ | ✓ | ✓ | ✓ | ✓ | 🗶 |
| EU368637 | India/06 | BA | ✓ | 🗶 | 🗶 | 🗶 | 🗶 | 🗶 |
| EU368635 | India/05 | BA | ✓ | ✓ | ✓ | ✓ | ✓ | 🗶 |
| EU368634 | India/05 | BA | ✓ | 🗶 | 🗶 | 🗶 | 🗶 | 🗶 |
| EU368633 | India/05 | BA | ✓ | ✓ | ✓ | ✓ | ✓ | 🗶 |
| EU368631 | India/05 | BA | ✓ | 🗶 | 🗶 | 🗶 | 🗶 | 🗶 |
| EU368627 | India/05 | BA | ✓ | ✓ | ✓ | ✓ | ✓ | 🗶 |
| EU368626 | India/05 | BA | ✓ | 🗶 | 🗶 | 🗶 | 🗶 | 🗶 |
| EU368625 | India/05 | BA | ✓ | 🗶 | 🗶 | 🗶 | 🗶 | 🗶 |
| EU368624 | India/05 | BA | ✓ | 🗶 | 🗶 | 🗶 | 🗶 | 🗶 |
| EU368623 | India/05 | BA | ✓ | 🗶 | 🗶 | 🗶 | 🗶 | 🗶 |
| KF981465 | Israel/07 | BA9 | ✓ | ✓ | 🗶 | ✓ | ✓ | 🗶 |
| KF981463 | Israel/06 | BA9 | ✓ | ✓ | 🗶 | ✓ | ✓ | 🗶 |
| KF981460 | Israel/11 | BA9 | ✓ | ✓ | 🗶 | ✓ | ✓ | 🗶 |
| JX079989 | Vietnam/11 | BA9 | ✓ | ✓ | 🗶 | ✓ | ✓ | 🗶 |
| JX079988 | Vietnam/10 | BA9 | ✓ | ✓ | 🗶 | ✓ | ✓ | 🗶 |
| JX079987 | Vietnam/10 | BA9 | ✓ | ✓ | 🗶 | ✓ | ✓ | 🗶 |
| JX079986 | Vietnam/10 | BA9 | ✓ | ✓ | 🗶 | ✓ | ✓ | 🗶 |
| JX079985 | Vietnam/10 | BA9 | ✓ | ✓ | 🗶 | ✓ | ✓ | 🗶 |
| JX079984 | Vietnam/10 | BA9 | ✓ | ✓ | 🗶 | ✓ | ✓ | 🗶 |
| JX079983 | Vietnam/10 | BA9 | ✓ | 🗶 | 🗶 | 🗶 | 🗶 | 🗶 |
| JX079982 | Vietnam/10 | BA9 | ✓ | ✓ | 🗶 | ✓ | ✓ | 🗶 |
| JX079981 | Vietnam/10 | BA9 | ✓ | ✓ | 🗶 | ✓ | ✓ | 🗶 |
| JX079980 | Vietnam/10 | BA9 | ✓ | ✓ | 🗶 | ✓ | ✓ | 🗶 |
| JX079979 | Vietnam/10 | BA9 | ✓ | ✓ | 🗶 | ✓ | ✓ | 🗶 |
| JX079978 | Vietnam/10 | BA9 | ✓ | 🗶 | 🗶 | 🗶 | 🗶 | 🗶 |
| JX079977 | Vietnam/10 | BA9 | ✓ | ✓ | 🗶 | ✓ | ✓ | 🗶 |
| JX079976 | Vietnam/10 | BA9 | ✓ | ✓ | 🗶 | ✓ | ✓ | 🗶 |
| JX079975 | Vietnam/10 | BA9 | ✓ | 🗶 | 🗶 | 🗶 | 🗶 | 🗶 |
| KJ939929 | Vietnam/10 | BA9 | ✓ | ✓ | 🗶 | ✓ | ✓ | 🗶 |
| KJ939928 | Vietnam/10 | BA9 | ✓ | 🗶 | 🗶 | 🗶 | 🗶 | 🗶 |
| KJ939927 | Vietnam/10 | BA9 | ✓ | ✓ | 🗶 | ✓ | ✓ | 🗶 |
| KJ939925 | Vietnam/09 | BA9 | ✓ | 🗶 | 🗶 | 🗶 | 🗶 | 🗶 |
| KJ939924 | Vietnam/09 | BA9 | ✓ | 🗶 | 🗶 | 🗶 | 🗶 | 🗶 |
| KJ939923 | Vietnam/09 | BA9 | ✓ | 🗶 | 🗶 | 🗶 | 🗶 | 🗶 |
| KJ939921 | Vietnam/09 | BA9 | ✓ | ✓ | 🗶 | ✓ | ✓ | 🗶 |
| KJ939934 | Vietnam/10 | BA9 | ✓ | 🗶 | 🗶 | 🗶 | 🗶 | 🗶 |
| KJ939933 | Vietnam/10 | BA9 | ✓ | ✓ | 🗶 | ✓ | ✓ | 🗶 |
| KJ939932 | Vietnam/10 | BA9 | ✓ | 🗶 | 🗶 | 🗶 | 🗶 | 🗶 |
| HQ699309 | Korea/10 | BA9 | ✓ | ✓ | 🗶 | ✓ | ✓ | 🗶 |
| HQ699303 | Korea/09 | BA9 | ✓ | ✓ | 🗶 | ✓ | ✓ | 🗶 |
| HQ699298 | Korea/08 | BA9 | ✓ | ✓ | 🗶 | ✓ | ✓ | 🗶 |
| HQ699290 | Korea/10 | BA9 | ✓ | ✓ | 🗶 | ✓ | ✓ | 🗶 |
| HQ699287 | Korea/10 | BA9 | ✓ | 🗶 | 🗶 | 🗶 | 🗶 | 🗶 |
| JN119970 | Cambodia/08 | BA9 | ✓ | ✓ | 🗶 | ✓ | ✓ | 🗶 |
| JN119993 | Cambodia/09 | BA9 | ✓ | ✓ | 🗶 | ✓ | ✓ | 🗶 |
| KC297484 | China/08 | BA9 | ✓ | ✓ | 🗶 | ✓ | ✓ | 🗶 |
| KC297482 | China/08 | BA9 | ✓ | ✓ | 🗶 | ✓ | ✓ | 🗶 |
| KC297480 | China/07 | BA9 | ✓ | ✓ | 🗶 | ✓ | ✓ | 🗶 |
| KC297476 | China/12 | BA9 | ✓ | ✓ | 🗶 | ✓ | ✓ | 🗶 |
| KC297475 | China/12 | BA9 | ✓ | ✓ | 🗶 | ✓ | ✓ | 🗶 |
| KC297473 | China/12 | BA9 | ✓ | ✓ | 🗶 | ✓ | ✓ | 🗶 |
| KC297469 | China/11 | BA9 | ✓ | ✓ | 🗶 | ✓ | ✓ | 🗶 |
| KC297465 | China/10 | BA9 | ✓ | ✓ | 🗶 | ✓ | ✓ | 🗶 |
| KC297461 | China/10 | BA9 | ✓ | ✓ | 🗶 | ✓ | ✓ | 🗶 |
| KC297459 | China/10 | BA9 | ✓ | ✓ | 🗶 | ✓ | ✓ | 🗶 |
| KC297452 | China/10 | BA9 | ✓ | 🗶 | 🗶 | 🗶 | 🗶 | 🗶 |
| KC297448 | China/10 | BA9 | ✓ | ✓ | 🗶 | ✓ | ✓ | 🗶 |
| KC297447 | China/10 | BA9 | ✓ | ✓ | 🗶 | ✓ | ✓ | 🗶 |
| KC297445 | China/10 | BA9 | ✓ | ✓ | 🗶 | ✓ | ✓ | 🗶 |
| KC297443 | China/09 | BA9 | ✓ | ✓ | 🗶 | ✓ | ✓ | 🗶 |
| KC297441 | China/09 | BA9 | ✓ | ✓ | 🗶 | ✓ | ✓ | 🗶 |
| KC297442 | China/09 | BA9 | ✓ | ✓ | 🗶 | ✓ | ✓ | 🗶 |
| KC297440 | China/09 | BA9 | ✓ | ✓ | 🗶 | ✓ | ✓ | 🗶 |
| KC297438 | China/09 | BA9 | ✓ | ✓ | 🗶 | ✓ | ✓ | 🗶 |
| KC297434 | China/09 | BA9 | ✓ | ✓ | 🗶 | ✓ | ✓ | 🗶 |
| KC297431 | China/09 | BA9 | ✓ | 🗶 | 🗶 | 🗶 | 🗶 | 🗶 |
| KC297424 | China/09 | BA9 | ✓ | ✓ | 🗶 | ✓ | ✓ | 🗶 |
| KC297423 | China/09 | BA9 | ✓ | ✓ | 🗶 | ✓ | ✓ | 🗶 |
| KC297422 | China/09 | BA9 | ✓ | ✓ | 🗶 | ✓ | ✓ | 🗶 |
| KC297457 | China/10 | BA9 | ✓ | ✓ | 🗶 | ✓ | ✓ | 🗶 |
| KC297464 | China/10 | BA9 | ✓ | 🗶 | 🗶 | 🗶 | 🗶 | 🗶 |
| KC477096 | S.Africa/12 | BA9 | ✓ | 🗶 | 🗶 | 🗶 | 🗶 | 🗶 |
| KC477090 | S.Africa/12 | BA9 | ✓ | ✓ | 🗶 | ✓ | ✓ | 🗶 |
| KC477085 | S.Africa/12 | BA9 | ✓ | ✓ | 🗶 | ✓ | ✓ | 🗶 |
| KC477080 | S.Africa/11 | BA9 | ✓ | 🗶 | 🗶 | 🗶 | 🗶 | 🗶 |
| KC477076 | S.Africa/11 | BA9 | ✓ | ✓ | 🗶 | ✓ | ✓ | 🗶 |
| KC477042 | S.Africa/11 | BA9 | ✓ | ✓ | 🗶 | ✓ | ✓ | 🗶 |
| KC477035 | S.Africa/11 | BA9 | ✓ | ✓ | 🗶 | ✓ | ✓ | 🗶 |
| KC477034 | S.Africa/11 | BA9 | ✓ | ✓ | 🗶 | ✓ | ✓ | 🗶 |
| KC477028 | S.Africa/11 | BA9 | ✓ | 🗶 | 🗶 | 🗶 | 🗶 | 🗶 |
| KC477014 | S.Africa/11 | BA9 | ✓ | ✓ | 🗶 | ✓ | ✓ | 🗶 |
| KC476998 | S.Africa/10 | BA9 | ✓ | ✓ | 🗶 | ✓ | ✓ | 🗶 |
| KC476993 | S.Africa/10 | BA9 | ✓ | ✓ | 🗶 | ✓ | ✓ | 🗶 |
| KC476966 | S.Africa/09 | BA9 | ✓ | 🗶 | 🗶 | 🗶 | 🗶 | 🗶 |
| KC476960 | S.Africa/09 | BA9 | ✓ | ✓ | 🗶 | ✓ | ✓ | 🗶 |
| KC476954 | S.Africa/09 | BA9 | ✓ | ✓ | 🗶 | ✓ | ✓ | 🗶 |
| KC476941 | S.Africa/09 | BA9 | ✓ | ✓ | 🗶 | ✓ | ✓ | 🗶 |
| EF219431 | S.Africa/06 | BA9 | ✓ | 🗶 | 🗶 | 🗶 | 🗶 | 🗶 |
| HQ711811 | S.Africa/09 | BA9 | ✓ | ✓ | 🗶 | ✓ | ✓ | 🗶 |
| HQ711799 | S.Africa/07 | BA9 | ✓ | ✓ | 🗶 | ✓ | ✓ | 🗶 |
| HQ711800 | S.Africa/06 | BA9 | ✓ | ✓ | 🗶 | ✓ | ✓ | 🗶 |
| KF437513 | Pakistan/12 | BA | ✓ | 🗶 | 🗶 | 🗶 | 🗶 | 🗶 |
| KF437512 | Pakistan/12 | BA | ✓ | ✓ | 🗶 | ✓ |  | 🗶 |
| KF437511 | Pakistan/12 | BA | ✓ | ✓ | 🗶 | ✓ |  | 🗶 |
| JQ933973 | Malaysia/09 | BA9 | ✓ | ✓ | 🗶 | ✓ | ✓ | 🗶 |
| JQ933972 | Malaysia/09 | BA9 | ✓ | ✓ | 🗶 | ✓ | ✓ | 🗶 |
| JQ933971 | Malaysia/09 | BA9 | ✓ | ✓ | 🗶 | ✓ | ✓ | 🗶 |
| JX256993 | Malaysia/06 | BA9 | ✓ | ✓ | 🗶 | ✓ | ✓ | 🗶 |
| AB749760 | Philippines/11 | BA9 | ✓ | 🗶 | 🗶 | 🗶 | 🗶 | 🗶 |
| AB749759 | Philippines/11 | BA9 | ✓ | ✓ | 🗶 | ✓ | ✓ | 🗶 |
| AB749758 | Philippines/11 | BA9 | ✓ | 🗶 | 🗶 | 🗶 | 🗶 | 🗶 |
| AB749755 | Philippines/11 | BA9 | ✓ | 🗶 | 🗶 | 🗶 | 🗶 | 🗶 |
| AB749754 | Philippines/11 | BA9 | ✓ | 🗶 | 🗶 | 🗶 | 🗶 | 🗶 |
| AB749753 | Philippines/11 | BA9 | ✓ | 🗶 | 🗶 | 🗶 | 🗶 | 🗶 |
| AB749752 | Philippines/11 | BA9 | ✓ | ✓ | 🗶 | ✓ | ✓ | 🗶 |
| AB749749 | Philippines/11 | BA9 | ✓ | ✓ | 🗶 | ✓ | ✓ | 🗶 |
| AB749746 | Philippines/11 | BA9 | ✓ | 🗶 | 🗶 | 🗶 | 🗶 | 🗶 |
| AB749745 | Philippines/11 | BA9 | ✓ | 🗶 | 🗶 | 🗶 | 🗶 | 🗶 |
| AB749743 | Philippines/11 | BA9 | ✓ | ✓ | 🗶 | ✓ | ✓ | 🗶 |
| AB749741 | Philippines/11 | BA9 | ✓ | 🗶 | 🗶 | 🗶 | 🗶 | 🗶 |
| AB749738 | Philippines/11 | BA9 | ✓ | 🗶 | 🗶 | 🗶 | 🗶 | 🗶 |
| AB749742 | Philippines/11 | BA9 | ✓ | ✓ | 🗶 | ✓ | ✓ | 🗶 |
| AB749737 | Philippines/11 | BA9 | ✓ | ✓ | 🗶 | ✓ | ✓ | 🗶 |
| AB749736 | Philippines/11 | BA9 | ✓ | ✓ | 🗶 | ✓ | ✓ | 🗶 |
| AB749735 | Philippines/11 | BA9 | ✓ | ✓ | 🗶 | ✓ | ✓ | 🗶 |
| AB749734 | Philippines/11 | BA9 | ✓ | ✓ | 🗶 | ✓ | ✓ | 🗶 |
| AB749732 | Philippines/11 | BA9 | ✓ | 🗶 | 🗶 | 🗶 | 🗶 | 🗶 |
| AB749731 | Philippines/11 | BA9 | ✓ | 🗶 | 🗶 | 🗶 | 🗶 | 🗶 |
| AB749730 | Philippines/11 | BA9 | ✓ | 🗶 | 🗶 | 🗶 | 🗶 | 🗶 |
| AB749729 | Philippines/11 | BA9 | ✓ | 🗶 | 🗶 | 🗶 | 🗶 | 🗶 |
| AB749728 | Philippines/11 | BA9 | ✓ | 🗶 | 🗶 | 🗶 | 🗶 | 🗶 |
| AB749727 | Philippines/11 | BA9 | ✓ | 🗶 | 🗶 | 🗶 | 🗶 | 🗶 |
| AB749725 | Philippines/11 | BA9 | ✓ | 🗶 | 🗶 | 🗶 | 🗶 | 🗶 |
| AB749723 | Philippines/11 | BA9 | ✓ | 🗶 | 🗶 | 🗶 | 🗶 | 🗶 |
| AB749719 | Philippines/11 | BA9 | ✓ | 🗶 | 🗶 | 🗶 | 🗶 | 🗶 |
| AB749718 | Philippines/11 | BA9 | ✓ | 🗶 | 🗶 | 🗶 | 🗶 | 🗶 |
| AB749717 | Philippines/11 | BA9 | ✓ | 🗶 | 🗶 | 🗶 | 🗶 | 🗶 |
| AB749716 | Philippines/10 | BA9 | ✓ | 🗶 | 🗶 | 🗶 | 🗶 | 🗶 |
| AB749715 | Philippines/10 | BA9 | ✓ | 🗶 | 🗶 | 🗶 | 🗶 | 🗶 |
| AB749714 | Philippines/10 | BA9 | ✓ | ✓ | 🗶 | ✓ | ✓ | 🗶 |
| AB749713 | Philippines/10 | BA9 | ✓ | 🗶 | 🗶 | 🗶 | 🗶 | 🗶 |
| AB749712 | Philippines/10 | BA9 | ✓ | 🗶 | 🗶 | 🗶 | 🗶 | 🗶 |
| AB749711 | Philippines/10 | BA9 | ✓ | 🗶 | 🗶 | 🗶 | 🗶 | 🗶 |
| AB749710 | Philippines/10 | BA9 | ✓ | ✓ | 🗶 | ✓ | ✓ | 🗶 |
| AB749709 | Philippines/10 | BA9 | ✓ | ✓ | 🗶 | ✓ | ✓ | 🗶 |
| AB749708 | Philippines/10 | BA9 | ✓ | 🗶 | 🗶 | 🗶 | 🗶 | 🗶 |
| AB749707 | Philippines/10 | BA9 | ✓ | ✓ | 🗶 | ✓ | ✓ | 🗶 |
| AB749706 | Philippines/10 | BA9 | ✓ | 🗶 | 🗶 | 🗶 | 🗶 | 🗶 |
| AB749705 | Philippines/10 | BA9 | ✓ | 🗶 | 🗶 | 🗶 | 🗶 | 🗶 |
| AB749704 | Philippines/10 | BA9 | ✓ | 🗶 | 🗶 | 🗶 | 🗶 | 🗶 |
| AB749703 | Philippines/10 | BA9 | ✓ | ✓ | 🗶 | ✓ | ✓ | 🗶 |
| AB749701 | Philippines/10 | BA9 | ✓ | 🗶 | 🗶 | 🗶 | 🗶 | 🗶 |
| AB749702 | Philippines/10 | BA9 | ✓ | ✓ | 🗶 | ✓ | ✓ | 🗶 |
| AB749700 | Philippines/10 | BA9 | ✓ | ✓ | 🗶 | ✓ | ✓ | 🗶 |
| AB749699 | Philippines/10 | BA9 | ✓ | 🗶 | 🗶 | 🗶 | 🗶 | 🗶 |
| AB749698 | Philippines/10 | BA9 | ✓ | 🗶 | 🗶 | 🗶 | 🗶 | 🗶 |
| AB749697 | Philippines/10 | BA9 | ✓ | ✓ | 🗶 | ✓ | ✓ | 🗶 |
| AB749696 | Philippines/10 | BA9 | ✓ | ✓ | 🗶 | ✓ | ✓ | 🗶 |
| AB749695 | Philippines/10 | BA9 | ✓ | ✓ | 🗶 | ✓ | ✓ | 🗶 |
| AB749721 | Philippines/11 | BA9 | ✓ | 🗶 | 🗶 | 🗶 | 🗶 | 🗶 |
| AB749694 | Philippines/09 | BA9 | ✓ | ✓ | 🗶 | ✓ | ✓ | 🗶 |
| AB749739 | Philippines/11 | BA9 | ✓ | 🗶 | 🗶 | 🗶 | 🗶 | 🗶 |
| KM873510 | Philippines/13 | BA9 | ✓ | ✓ | 🗶 | ✓ | ✓ | 🗶 |
| KM873509 | Philippines/13 | BA9 | ✓ | ✓ | 🗶 | ✓ | ✓ | 🗶 |
| KM873503 | Philippines/13 | BA9 | ✓ | ✓ | 🗶 | ✓ | ✓ | 🗶 |
| KM873508 | Philippines/13 | BA9 | ✓ | ✓ | 🗶 | ✓ | ✓ | 🗶 |
| KM873502 | Philippines/13 | BA9 | ✓ | ✓ | 🗶 | ✓ | ✓ | 🗶 |
| KM873501 | Philippines/13 | BA9 | ✓ | ✓ | 🗶 | ✓ | ✓ | 🗶 |
| KM873500 | Philippines/13 | BA9 | ✓ | ✓ | 🗶 | ✓ | ✓ | 🗶 |
| KM873497 | Philippines/13 | BA9 | ✓ | ✓ | 🗶 | ✓ | ✓ | 🗶 |
| KM873498 | Philippines/13 | BA9 | ✓ | ✓ | 🗶 | ✓ | ✓ | 🗶 |
| KM873496 | Philippines/13 | BA9 | ✓ | ✓ | 🗶 | ✓ | ✓ | 🗶 |
| KM873507 | Philippines/12 | BA9 | ✓ | ✓ | 🗶 | ✓ | ✓ | 🗶 |
| KM873506 | Philippines/12 | BA9 | ✓ | 🗶 | 🗶 | 🗶 | 🗶 | 🗶 |
| KM873504 | Philippines/12 | BA9 | ✓ | ✓ | 🗶 | ✓ | ✓ | 🗶 |
| KM873463 | Philippines/13 | BA9 | ✓ | ✓ | 🗶 | ✓ | ✓ | 🗶 |
| KM873462 | Philippines/13 | BA9 | ✓ | 🗶 | 🗶 | 🗶 | 🗶 | 🗶 |
| KM873461 | Philippines/12 | BA9 | ✓ | ✓ | 🗶 | ✓ | ✓ | 🗶 |
| DQ227395 | Argentina/04 | BA9 | ✓ | ✓ | 🗶 | ✓ | ✓ | 🗶 |
| JX576752 | Netherlands/09 | GB13 | ✓ | ✓ | 🗶 | ✓ | ✓ | 🗶 |
| JX576744 | Netherlands/08 | GB13 | ✓ | ✓ | 🗶 | ✓ | ✓ | 🗶 |
| JX576743 | Netherlands/09 | GB13 | ✓ | ✓ | 🗶 | ✓ | ✓ | 🗶 |
| JX576738 | Netherlands/10 | GB13 | ✓ | ✓ | 🗶 | ✓ | ✓ | 🗶 |
| JX576735 | Netherlands/12 | GB13 | ✓ | ✓ | 🗶 | ✓ | ✓ | 🗶 |
| KM402755 | Spain/13 | BA9 | ✓ | ✓ | 🗶 | ✓ | ✓ | 🗶 |
| KM402748 | Spain/14 | BA9 | ✓ | ✓ | 🗶 | ✓ | ✓ | 🗶 |
| KM402745 | Spain/14 | BA9 | ✓ | ✓ | 🗶 | ✓ | ✓ | 🗶 |
| KM402744 | Spain/14 | BA9 | ✓ | ✓ | 🗶 | ✓ | ✓ | 🗶 |
| KM402742 | Spain/14 | BA9 | ✓ | ✓ | 🗶 | ✓ | ✓ | 🗶 |
| KM402738 | Spain/13 | BA9 | ✓ | ✓ | 🗶 | ✓ | ✓ | 🗶 |
| KM402735 | Spain/13 | BA9 | ✓ | ✓ | 🗶 | ✓ | ✓ | 🗶 |
| KM402732 | Spain/14 | BA9 | ✓ | ✓ | 🗶 | ✓ | ✓ | 🗶 |
| KM402726 | Spain/14 | BA9 | ✓ | ✓ | 🗶 | ✓ | ✓ | 🗶 |
| KM402728 | Spain/14 | BA9 | ✓ | ✓ | 🗶 | ✓ | ✓ | 🗶 |
| KM402721 | Spain/14 | BA9 | ✓ | ✓ | 🗶 | ✓ | ✓ | 🗶 |
| KM402720 | Spain/14 | BA9 | ✓ | 🗶 | 🗶 | 🗶 | 🗶 | 🗶 |
| KM402719 | Spain/14 | BA9 | ✓ | ✓ | 🗶 | ✓ | ✓ | 🗶 |
| KM402717 | Spain/14 | BA9 | ✓ | 🗶 | 🗶 | 🗶 | 🗶 | 🗶 |
| KM402713 | Spain/14 | BA9 | ✓ | ✓ | 🗶 | ✓ | ✓ | 🗶 |
| KM402711 | Spain/13 | BA9 | ✓ | ✓ | 🗶 | ✓ | ✓ | 🗶 |
| KM402710 | Spain/14 | BA9 | ✓ | ✓ | 🗶 | ✓ | ✓ | 🗶 |
| KM402707 | Spain/13 | BA9 | ✓ | ✓ | 🗶 | ✓ | ✓ | 🗶 |
| KM402698 | Spain/13 | BA9 | ✓ | ✓ | 🗶 | ✓ | ✓ | 🗶 |
| KM402689 | Spain/13 | BA9 | ✓ | 🗶 | 🗶 | 🗶 | 🗶 | 🗶 |
| KM402687 | Spain/13 | BA9 | ✓ | ✓ | 🗶 | ✓ | ✓ | 🗶 |
| KM402694 | Spain/13 | BA9 | ✓ | 🗶 | 🗶 | 🗶 | 🗶 | 🗶 |
| KM402684 | Spain/13 | BA9 | ✓ | ✓ | 🗶 | ✓ | ✓ | 🗶 |
| KM402683 | Spain/13 | BA9 | ✓ | ✓ | 🗶 | ✓ | ✓ | 🗶 |
| KM402675 | Spain/14 | BA9 | ✓ | ✓ | 🗶 | ✓ | ✓ | 🗶 |
| HM459882 | Japan/09 | BA9 | ✓ | 🗶 | 🗶 | 🗶 | 🗶 | 🗶 |
| HM459880 | Japan/09 | BA9 | ✓ | ✓ | 🗶 | ✓ | ✓ | 🗶 |
| HM459879 | Japan/07 | BA9 | ✓ | 🗶 | 🗶 | 🗶 | 🗶 | 🗶 |
| HM459878 | Japan/07 | BA9 | ✓ | 🗶 | 🗶 | 🗶 | 🗶 | 🗶 |
| HM459877 | Japan/06 | BA9 | ✓ | 🗶 | 🗶 | 🗶 | 🗶 | 🗶 |
| HM459876 | Japan/06 | BA9 | ✓ | ✓ | 🗶 | ✓ | ✓ | 🗶 |
| HM459881 | Japan/09 | BA9 | ✓ | 🗶 | 🗶 | 🗶 | 🗶 | 🗶 |
| AB603469 | Japan/06 | BA9 | ✓ | ✓ | 🗶 | ✓ | ✓ | 🗶 |
| AB603467 | Japan/06 | BA9 | ✓ | 🗶 | 🗶 | 🗶 | 🗶 | 🗶 |
| AB603470 | Japan/05 | BA9 | ✓ | ✓ | 🗶 | ✓ | ✓ | 🗶 |
| AB551099 | Japan/07 | BA9 | ✓ | ✓ | 🗶 | ✓ | ✓ | 🗶 |
| AB683224 | Japan/09 | BA9 | ✓ | ✓ | 🗶 | ✓ | ✓ | 🗶 |
| AB683227 | Japan/10 | BA9 | ✓ | ✓ | 🗶 | ✓ | ✓ | 🗶 |
| AB683228 | Japan/10 | BA9 | ✓ | ✓ | 🗶 | ✓ | ✓ | 🗶 |
| AB683230 | Japan/10 | BA9 | ✓ | ✓ | 🗶 | ✓ | ✓ | 🗶 |
| AB683237 | Japan/10 | BA9 | ✓ | ✓ | 🗶 | ✓ | ✓ | 🗶 |
| AB775991 | Japan/10 | BA9 | ✓ | ✓ | 🗶 | ✓ | ✓ | 🗶 |
| AB775993 | Japan/10 | BA9 | ✓ | ✓ | 🗶 | ✓ | ✓ | 🗶 |
| AB775997 | Japan/10 | BA9 | ✓ | ✓ | 🗶 | ✓ | ✓ | 🗶 |
| AB775999 | Japan/11 | BA9 | ✓ | ✓ | 🗶 | ✓ | ✓ | 🗶 |
| LC060589 | Japan/14 | BA9 | ✓ | 🗶 | 🗶 | 🗶 | 🗶 | 🗶 |
| LC060588 | Japan/14 | BA9 | ✓ | ✓ | 🗶 | ✓ | ✓ | 🗶 |
| LC060587 | Japan/14 | BA9 | ✓ | ✓ | 🗶 | ✓ | ✓ | 🗶 |
| LC060586 | Japan/13 | BA9 | ✓ | ✓ | 🗶 | ✓ | ✓ | 🗶 |
| LC060585 | Japan/13 | BA9 | ✓ | ✓ | 🗶 | ✓ | ✓ | 🗶 |
| LC060584 | Japan/14 | BA9 | ✓ | ✓ | 🗶 | ✓ | ✓ | 🗶 |
| LC060578 | Japan/13 | BA9 | ✓ | ✓ | 🗶 | ✓ | ✓ | 🗶 |
| LC060577 | Japan/13 | BA9 | ✓ | ✓ | 🗶 | ✓ | ✓ | 🗶 |
| LC060576 | Japan/13 | BA9 | ✓ | ✓ | 🗶 | ✓ | ✓ | 🗶 |
| LC060575 | Japan/11 | BA9 | ✓ | ✓ | 🗶 | ✓ | ✓ | 🗶 |
| LC060574 | Japan/14 | BA9 | ✓ | ✓ | 🗶 | ✓ | ✓ | 🗶 |
| LC060573 | Japan/13 | BA9 | ✓ | ✓ | 🗶 | ✓ | ✓ | 🗶 |
| LC060572 | Japan/13 | BA9 | ✓ | ✓ | 🗶 | ✓ | ✓ | 🗶 |
| LC060571 | Japan/14 | BA9 | ✓ | ✓ | 🗶 | ✓ | ✓ | 🗶 |
| LC060570 | Japan/14 | BA9 | ✓ | ✓ | 🗶 | ✓ | ✓ | 🗶 |
| LC060569 | Japan/14 | BA9 | ✓ | ✓ | 🗶 | ✓ | ✓ | 🗶 |
| LC060568 | Japan/14 | BA9 | ✓ | 🗶 | 🗶 | 🗶 | 🗶 | 🗶 |
| LC060567 | Japan/12 | BA9 | ✓ | ✓ | 🗶 | ✓ | ✓ | 🗶 |
| LC060566 | Japan/14 | BA9 | ✓ | ✓ | 🗶 | ✓ | ✓ | 🗶 |
| LC060565 | Japan/14 | BA9 | ✓ | ✓ | 🗶 | ✓ | ✓ | 🗶 |
| LC060564 | Japan/14 | BA9 | ✓ | ✓ | 🗶 | ✓ | ✓ | 🗶 |
| KJ710420 | Germany/13 | BA9 | ✓ | 🗶 | 🗶 | 🗶 | 🗶 | 🗶 |
| KJ710419 | Germany/13 | BA9 | ✓ | ✓ | 🗶 | ✓ | ✓ | 🗶 |
| KJ710418 | Germany/13 | BA9 | ✓ | ✓ | 🗶 | ✓ | ✓ | 🗶 |
| KJ710417 | Germany/13 | BA9 | ✓ | ✓ | 🗶 | ✓ | ✓ | 🗶 |
| KJ710416 | Germany/13 | BA9 | ✓ | ✓ | 🗶 | ✓ | ✓ | 🗶 |
| KJ710414 | Germany/13 | BA9 | ✓ | ✓ | 🗶 | ✓ | ✓ | 🗶 |
| KJ710412 | Germany/13 | BA9 | ✓ | ✓ | 🗶 | ✓ | ✓ | 🗶 |
| KJ710413 | Germany/13 | BA9 | ✓ | ✓ | 🗶 | ✓ | ✓ | 🗶 |
| KJ710409 | Germany/13 | BA9 | ✓ | 🗶 | 🗶 | 🗶 | 🗶 | 🗶 |
| KJ710407 | Germany/13 | BA9 | ✓ | ✓ | 🗶 | ✓ | ✓ | 🗶 |
| JX967575 | Germany/12 | BA9 | ✓ | ✓ | 🗶 | ✓ | ✓ | 🗶 |
| KC342326 | Thiland/09 | BA9 | ✓ | ✓ | 🗶 | ✓ | ✓ | 🗶 |
| KC342344 | Thiland/11 | BA9 | ✓ | ✓ | 🗶 | ✓ | ✓ | 🗶 |
| KC342345 | Thiland/11 | BA9 | ✓ | ✓ | 🗶 | ✓ | ✓ | 🗶 |
| KC342347 | Thiland/11 | BA9 | ✓ | ✓ | 🗶 | ✓ | ✓ | 🗶 |
| KC342342 | Thiland/11 | BA9 | ✓ | 🗶 | 🗶 | 🗶 | 🗶 | 🗶 |
| KC342341 | Thiland/11 | BA9 | ✓ | 🗶 | 🗶 | 🗶 | 🗶 | 🗶 |
| KC342340 | Thiland/10 | BA9 | ✓ | ✓ | 🗶 | ✓ | ✓ | 🗶 |
| KC342339 | Thiland/10 | BA9 | ✓ | ✓ | 🗶 | ✓ | ✓ | 🗶 |
| KC342338 | Thiland/10 | BA9 | ✓ | ✓ | 🗶 | ✓ | ✓ | 🗶 |
| KC342337 | Thiland/10 | BA9 | ✓ | 🗶 | 🗶 | 🗶 | 🗶 | 🗶 |
| KC342334 | Thiland/10 | BA9 | ✓ | 🗶 | 🗶 | 🗶 | 🗶 | 🗶 |
| KC342333 | Thiland/10 | BA9 | ✓ | 🗶 | 🗶 | 🗶 | 🗶 | 🗶 |
| KC342335 | Thiland/10 | BA9 | ✓ | 🗶 | 🗶 | 🗶 | 🗶 | 🗶 |
| KF156570 | Kenya/08 | BA | ✓ | ✓ | 🗶 | ✓ | 🗶 | 🗶 |
| KP862526 | Kenya/12 | BA | ✓ | ✓ | 🗶 | ✓ | 🗶 | 🗶 |
| KP862466 | Kenya/12 | BA | ✓ | ✓ | 🗶 | ✓ | 🗶 | 🗶 |
| KP862450 | Kenya/11 | BA | ✓ | ✓ | 🗶 | ✓ | 🗶 | 🗶 |
| JQ806031 | Brazil/07 | BA9 | ✓ | ✓ | 🗶 | ✓ | ✓ | 🗶 |
| JQ806044 | Brazil/07 | BA9 | ✓ | ✓ | 🗶 | ✓ | ✓ | 🗶 |
| JQ806050 | Brazil/06 | BA9 | ✓ | ✓ | 🗶 | ✓ | ✓ | 🗶 |
| EU582474 | Brazil/05 | BA9 | ✓ | ✓ | 🗶 | ✓ | ✓ | 🗶 |
| JQ844871 | Croatia/06 | BA9 | ✓ | ✓ | 🗶 | ✓ | ✓ | 🗶 |
| JQ844868 | Croatia/07 | BA9 | ✓ | ✓ | 🗶 | ✓ | ✓ | 🗶 |
| HM778070 | Ireland/04 | BA | ✓ | ✓ | 🗶 | ✓ | ✓ | 🗶 |
| HM778078 | Ireland/05 | BA | ✓ | ✓ | 🗶 | ✓ | ✓ | 🗶 |
| HM778089 | Ireland/09 | BA | ✓ | ✓ | 🗶 | ✓ | ✓ | 🗶 |
| HM778076 | Ireland/07 | BA | ✓ | ✓ | 🗶 | ✓ | ✓ | 🗶 |
| HM778067 | Ireland/06 | BA | ✓ | ✓ | 🗶 | ✓ | ✓ | 🗶 |
| HM778084 | Ireland/09 | BA | ✓ | ✓ | 🗶 | ✓ | ✓ | 🗶 |
| HM778085 | Ireland/06 | BA | ✓ | 🗶 | 🗶 | 🗶 | 🗶 | 🗶 |
| KF171945 | Iran/13 | BA | ✓ | ✓ | 🗶 | ✓ | ✓ | 🗶 |
| HM063470 | Iran/09 | BA | ✓ | ✓ | 🗶 | ✓ | ✓ | 🗶 |
| HM063467 | Iran/09 | BA | ✓ | ✓ | 🗶 | ✓ | ✓ | 🗶 |
| HM063464 | Iran/09 | BA | ✓ | ✓ | 🗶 | ✓ | ✓ | 🗶 |
| HM063468 | Iran/09 | BA | ✓ | ✓ | 🗶 | ✓ | ✓ | 🗶 |
| HM063463 | Iran/09 | BA | ✓ | 🗶 | 🗶 | 🗶 | 🗶 | 🗶 |
| KU726061 | S.Arabia/14 | BA9 | ✓ | 🗶 | 🗶 | 🗶 | 🗶 | 🗶 |
| KU726062 | S.Arabia/14 | BA9 | ✓ | 🗶 | 🗶 | 🗶 | 🗶 | 🗶 |
| KU726063 | S.Arabia/14 | BA9 | ✓ | 🗶 | 🗶 | 🗶 | 🗶 | 🗶 |
| KU726064 | S.Arabia/14 | BA9 | ✓ | 🗶 | 🗶 | 🗶 | 🗶 | 🗶 |
| KU726065 | S.Arabia/14 | BA9 | ✓ | 🗶 | 🗶 | 🗶 | 🗶 | 🗶 |
| JX198151 | USA/05 | BA9 | ✓ | ✓ | 🗶 | ✓ | ✓ | 🗶 |
| JX198154 | USA/04 | BA | ✓ | 🗶 | 🗶 | 🗶 | 🗶 | 🗶 |
| JX198153 | USA/04 | BA | ✓ | 🗶 | 🗶 | 🗶 | 🗶 | 🗶 |
| KP284744 | Italy/14 | BA9 | ✓ | ✓ | 🗶 | ✓ | ✓ | 🗶 |
| KP284746 | Italy/13 | BA9 | ✓ | ✓ | 🗶 | ✓ | ✓ | 🗶 |
| KP284740 | Italy/14 | BA9 | ✓ | ✓ | 🗶 | ✓ | ✓ | 🗶 |
| KP284735 | Italy/12 | BA9 | ✓ | ✓ | 🗶 | ✓ | ✓ | 🗶 |
| KP284734 | Italy/11 | BA9 | ✓ | ✓ | 🗶 | ✓ | ✓ | 🗶 |
| KP284728 | Italy/11 | BA9 | ✓ | ✓ | 🗶 | ✓ | ✓ | 🗶 |
| KP284726 | Italy/13 | BA9 | ✓ | ✓ | 🗶 | ✓ | ✓ | 🗶 |
| KP284722 | Italy/10 | BA9 | ✓ | ✓ | 🗶 | ✓ | ✓ | 🗶 |
| KP284721 | Italy/09 | BA9 | ✓ | ✓ | 🗶 | ✓ | ✓ | 🗶 |
| KP284715 | Italy/12 | BA9 | ✓ | ✓ | 🗶 | ✓ | ✓ | 🗶 |
| KP284714 | Italy/10 | BA9 | ✓ | ✓ | 🗶 | ✓ | ✓ | 🗶 |
| AB603478 | Japan/06 | BA8 | ✓ | ✓ | 🗶 | ✓ | ✓ | 🗶 |
| AB603476 | Japan/05 | BA7 | ✓ | ✓ | 🗶 | ✓ | ✓ | 🗶 |
| AY751110 | Belgium/02 | BA6 | ✓ | ✓ | 🗶 | ✓ | 🗶 | 🗶 |
| AB603483 | Japan/02 | BA5 | ✓ | ✓ | 🗶 | ✓ | 🗶 | 🗶 |
| AY927409 | Canada/01-02 | BA4 | ✓ | ✓ | 🗶 | ✓ | 🗶 | 🗶 |
| DQ227407 | Argentina/04 | BA4 | ✓ | ✓ | 🗶 | ✓ | 🗶 | 🗶 |
| DQ227387 | Argentina/02 | BA3 | ✓ | ✓ | 🗶 | ✓ | 🗶 | 🗶 |
| KF981461 | Israel/10 | BA2 | ✓ | ✓ | 🗶 | ✓ | 🗶 | 🗶 |
| AY751131 | Belgium/99 | BA1 | ✓ | ✓ | 🗶 | ✓ | 🗶 | 🗶 |
| AY333364 | Argentina/99 | BA1 | ✓ | ✓ | 🗶 | ✓ | 🗶 | ✓ |
| JN120007 | Cambodia/09 | SAB4 | ✓ | ✓ | 🗶 | ✓ | 🗶 | 🗶 |
| JN119987 | Cambodia/09 | SAB4 | ✓ | ✓ | 🗶 | ✓ | 🗶 | 🗶 |
| AY488800 | Uruguay/99 | SAB3 | ✓ | ✓ | 🗶 | ✓ | 🗶 | 🗶 |
| AF309678 | Mozambique/99 | SAB2 | ✓ | ✓ | 🗶 | ✓ | 🗶 | 🗶 |
| AY524573 | Kenya/02 | SAB1 | ✓ | ✓ | 🗶 | ✓ | 🗶 | 🗶 |
| AY751237 | Belgium/00 | GB6 | ✓ | ✓ | 🗶 | ✓ | 🗶 | 🗶 |
| AY751280 | Belgium/85 | GB5 | ✓ | ✓ | 🗶 | ✓ | 🗶 | 🗶 |
| AY672698 | Argentina/99 | GB4 | ✓ | ✓ | 🗶 | ✓ | 🗶 | 🗶 |
| AY672701 | Argentina/01 | GB3 | ✓ | ✓ | 🗶 | ✓ | 🗶 | 🗶 |
| DQ171849 | N.Zealand/88 | GB2 | ✓ | ✓ | 🗶 | ✓ | 🗶 | 🗶 |
| AF065250 | USA/90 | GB1 | ✓ | ✓ | 🗶 | ✓ | 🗶 | 🗶 |
| M17213 | USA/62 | Prototype | ✓ | ✓ | 🗶 | ✓ | 🗶 | 🗶 |

**✓** Denotes sequences used

**🗶** Denotes sequences not used

Red colour represents the study sequence
